# Mutation of *DEFECTIVE EMBRYO SAC1* results in a low seed-setting rate in rice by regulating embryo sac development

**DOI:** 10.1093/jxb/erac506

**Published:** 2023-01-18

**Authors:** Xia Hu, Ping Yu, Yingxin Zhang, Zhiqiang Gao, Bin Sun, Weixun Wu, Chenwei Deng, Adil Abbas, Yongbo Hong, Lianping Sun, Qunen Liu, Pao Xue, Beifang Wang, Xiaodeng Zhan, Liyong Cao, Shihua Cheng

**Affiliations:** China National Rice Research Institute, Hangzhou, Zhejiang, 311400, China; China National Rice Research Institute, Hangzhou, Zhejiang, 311400, China; China National Rice Research Institute, Hangzhou, Zhejiang, 311400, China; Gannan Normal University, Ganzhou, Jiangxi, 341000, China; Shanghai Academy of Agricultural Sciences, Shanghai, 201403, China; China National Rice Research Institute, Hangzhou, Zhejiang, 311400, China; Zhoukou Academy of Agricultural Sciences, Zhoukou, Henan, 466001, China; China National Rice Research Institute, Hangzhou, Zhejiang, 311400, China; China National Rice Research Institute, Hangzhou, Zhejiang, 311400, China; China National Rice Research Institute, Hangzhou, Zhejiang, 311400, China; China National Rice Research Institute, Hangzhou, Zhejiang, 311400, China; China National Rice Research Institute, Hangzhou, Zhejiang, 311400, China; China National Rice Research Institute, Hangzhou, Zhejiang, 311400, China; China National Rice Research Institute, Hangzhou, Zhejiang, 311400, China; China National Rice Research Institute, Hangzhou, Zhejiang, 311400, China; China National Rice Research Institute, Hangzhou, Zhejiang, 311400, China; University of Nottingham, UK

**Keywords:** Embryo sac, female sterile, fertilization, *OsDES1*, rice, seed-setting rate

## Abstract

The seed-setting rate has a significant effect on grain yield in rice (*Oryza sativa* L.). Embryo sac development is essential for seed setting; however, the molecular mechanism underlying this process remains unclear. Here, we isolated *defective embryo sac1* (*des1*), a rice mutant with a low seed-setting rate. Cytological examination showed degenerated embryo sacs and reduced fertilization capacity in *des1*. Map-based cloning revealed a nonsense mutation in *OsDES1*, a gene that encodes a putative nuclear envelope membrane protein (NEMP)-domain-containing protein that is preferentially expressed in pistils. The *OsDES1* mutation disrupts the normal formation of functional megaspores, which ultimately results in a degenerated embryo sac in *des1*. Reciprocal crosses showed that fertilization is abnormal and that the female reproductive organ is defective in *des1*. OsDES1 interacts with LONELY GUY (LOG), a cytokinin-activating enzyme that acts in the final step of cytokinin synthesis; mutation of *LOG* led to defective female reproductive organ development. These results demonstrate that *OsDES1* functions in determining the rice seed-setting rate by regulating embryo sac development and fertilization. Our study sheds light on the function of NEMP-type proteins in rice reproductive development.

## Teaser

*OsDES1*encodes a NEMP-domain protein that interacts with a cytokinin-activating enzyme, LOG, to determine the seed-setting rate in rice by regulating embryo sac development.

## Introduction

In addition to its role as a primary staple for more than half the world’s population, rice (*Oryza sativa* L.) serves as a model species for studies of plant development and biology in monocotyledons. Rice yield is typically determined by the grain weight, panicle number, number of grains per panicle, and seed-setting rate. In the past few decades, significant advances have been made in understanding the molecular mechanisms that control the grain weight, panicle number, and number of grains per panicle (Zuo *et al.*, 2014). Studies have shown that adverse environmental conditions can dramatically reduce the seed**-**setting rate in rice, resulting in serious yield reduction (Li *et al.*, 2013). Recent findings have shed light on the molecular mechanisms that regulate the seed**-**setting rate in rice, and several genes related to this trait, such as *PSS1*, *PTB1*, *OsSPX1*, *OsCNGC13*, *OsOAT*, *OsALMT7*, *DPS1*, *OsROS1*, *OsMLH3*, and *ESD1*, have been reported (Zhou *et al.*, 2011; Li *et al.*, 2013; Zhang *et al.*, 2016; Xu *et al.*, 2017; Heng *et al.*, 2018; Liu *et al.*, 2018; Zafar *et al.*, 2019; Xu *et al.*, 2020; Mao *et al.*, 2021; Wang *et al.*, 2021). The low seed-setting rate in *indica–japonica* hybrids has become a major roadblock that restricts further improvements in grain yield (Li *et al.*, 2016). Hence, more research is needed to explore the molecular mechanisms underlying seed setting in rice.

The seed**-**setting rate in rice is affected by many genetic and environmental factors, including defective embryo sac development, malformed floral organ morphology, disordered pollen grain formation, inadequate anther dehiscence, gametophytic incompatibility, and abnormal temperature (Xu *et al.*, 2017). Embryo sac development is vital to the correct functioning of steps in the reproductive process such as pollen tube guidance, double fertilization, induced seed development, and maternal control of seed development after fertilization (Ray *et al.*, 1997; Christensen *et al.*, 1998; Drews *et al.*, 1998; Yadegari and Drews, 2004). Embryo sac development is impacted by various physiological and environmental factors, such as plant hormones (Pischke *et al.*, 2002; Bencivenga *et al.*, 2012; Cheng *et al.*, 2013) and climatic conditions (Li *et al.*, 2013). Many genes controlling female reproductive organ development in plants have been studied. For example, Arabidopsis *WUS* and *SPL* and maize *MAC1* regulate the differentiation of somatic cells into germ cells (Sheridan *et al.*, 1996; Yang *et al.*, 1999; Lieber *et al.*, 2011). Several meiosis-related genes function in female fertility, including *SWI1* (Boateng *et al.*, 2008) and *ARP6* (Qin *et al.*, 2014) in Arabidopsis, and *PAIR1* (Nonomura *et al.*, 2004a), *PAIR2* (Nonomura *et al.*, 2004b), *PAIR3* (Yuan *et al.*, 2009), *OsRPA1a* (Chang *et al.*, 2009), *RAD51C* (Kou *et al.*, 2012), *OsMSH4* (Wang *et al.*, 2016), *OsSHOC1* (Ren *et al.*, 2019), *OsMFS1* (Lu *et al.*, 2020), and *OsMLH3* (Mao *et al.*, 2021) in rice. *MYB64* and *MYB119* (Rabiger and Drews, 2013) and *BLH1* (Pagnussat *et al.*, 2007) in Arabidopsis, and *OsAPC6* (Kumar *et al.*, 2010; Awasthi *et al.*, 2012) and *OsDEES1* (Wang *et al.*, 2012) in rice, are associated with mitosis and regulate the development of the embryo sac and seed. The cytokinin-activating enzyme encoded by *LONELY GUY* (*LOG*) is essential for ovule and pistil formation (Kurakawa *et al.*, 2007; Yamaki *et al.*, 2011). Recently, *OsROS1*, *ESD1*, and *OsMLH3* have been reported to function in rice embryo sac development (Xu *et al.*, 2020; Wang *et al.*, 2021; Mao *et al.*, 2021).

In most angiosperms, embryo sac development generally includes megasporogenesis and megagametogenesis. The megasporocyte develops into a seven-celled structure through two meiotic divisions and three consecutive mitotic divisions (Yadegari and Drews, 2004; Drews and Koltunow, 2011; Nakajima, 2018). These cells make up four groups that function in fertilization, embryogenesis, and nutrition of the embryo sac and embryo (Reiser and Fischer, 1993; Chen *et al.*, 2007; Li *et al.*, 2015; Higashiyama and Yang, 2017; Meng *et al.*, 2020; Sun *et al.*, 2021). The male and female gametes undergo fusion and develop into the embryo and endosperm. The integuments go through structural and biochemical specialization as the ovule forms into a seed (Robinson-Beers *et al.*, 1992; Li *et al.*, 2013; Dresselhaus *et al.*, 2016; Sankaranarayanan and Higashiyama, 2018). The embryo sac is embedded in sporophytic tissues of the ovule, making it difficult to directly isolate embryo sac tissue for research (Jones-Rhoades *et al.*, 2007). In the past two decades, due to the difficulty of mutant acquisition and morphological identification and the complexity of genetic mechanisms, progress in research on female sterility has lagged. Therefore, we decided to evaluate female-sterility-related genes and apply them to target developmental mutants in rice.

In this study, we report on the role of a putative nuclear envelope membrane protein (NEMP) domain-containing protein, DEFECTIVE EMBRYO SAC1 (OsDES1), in seed setting by regulating embryo sac development. The loss-of-function mutant *des1* displayed a significant reduction in seed-setting rate due to embryo sac degeneration and defective fertilization. We propose that the abnormal formation of functional megaspores in *des1* causes embryo sac degeneration and reduced fertility. Our study provides insights into the roles of *OsDES1* in female reproductive development and seed setting in rice.

## Materials and methods

### Plant materials and growth conditions

The rice plants used in this study were grown in paddy fields at the China National Rice Research Institute, Hangzhou, Zhejiang Province, and in Lingshui, Hainan Province, China. The *des1* mutant, which shows an abnormal seed-setting rate, was isolated from a ^60^Co-γ-radiation-induced mutant library of the *indica* rice cv. ‘Zhonghui8015’ (ZH8015). An F_2_ mapping population was derived from a cross between the *japonica* rice cv. 02428 and the homozygous *des1* mutant. The mutant plants showed genetic stability in both Zhejiang and Hainan.

### Preparation of embryo sacs

Wild-type (WT) and mutant spikelets were collected at different stages of embryo sac development based on the length of the floret. The spikelets were fixed in ethanol:formaldehyde:glacial acetic acid (18:1:1) solution (FAA) and vacuum infiltrated. The samples were kept in 70% ethanol for embryo sac observation. The spikelets were dissected in 70% ethanol, hydrated sequentially in 50% ethanol, 30% ethanol, and distilled water, and then transferred to 2% aluminum potassium sulfate for 20 min. The samples were stained with 10 mg l^–1^ eosin B solution dissolved in 4% sucrose overnight at room temperature, pretreated with 2% aluminum potassium sulfate for 20 min, and then washed with distilled water followed by dehydration using ethanol solutions at concentrations of 30, 50, 70, 90, and 100%. The samples were transferred into a mixture of methylsalicylate and absolute ethanol (1:1) for 1 h and then moved to 100% methylsalicylate solution for 8 h (Zhao *et al.*, 2007; Zeng *et al.*, 2009). Finally, the ovaries were imaged with a Zeiss LSM710 laser scanning confocal microscope.

### Histological analysis

The developmental stages of the rice anthers were identified as previously described (Zhang and Wilson, 2009; Zhang *et al.*, 2011). Spikelets at different developmental stages were selected and fixed in FAA for semi-thin sectioning. Anthers at different developmental stages were selected and embedded in a standard resin for semi-thin sectioning according to a previously published protocol (Li *et al.*, 2006). The samples were dehydrated in a graded ethanol series from 50% to 100% and embedded in Technovit 7100 resin (Heraeus, Kulzer, Germany), which was then allowed to solidify at 50 °C for 3–4 days. Transverse sections of 2 μm thickness were cut using a Leica RM2265 fully automated rotary microtome, stained with 0.25% toluidine blue O, and photographed under a Leica DM2000 light microscope. The observation of embryo sac development was performed as previously described (Yamaki *et al.*, 2011). All samples were dehydrated in a graded ethanol series, substituted with xylene and embedded in paraffin, then cut at 8 μm thickness, and finally stained with hematoxylin, hyalinized, and sealed. The sealed sections were imaged with a Zeiss LSM710 laser scanning confocal microscope.

### Acetocarmine and DAPI staining

For observations of microspore development, spikelet samples were chosen from the premeiotic to mature stages and fixed in FAA. The microspores from crushed anthers were stained with 1% (w/v) acetocarmine solution. After 2 min, microspores were observed under a Leica DM2000 light microscope. To observe the microspores of the mature stages better, DAPI (4ʹ,6-diamidino-2-phenylindole) staining was used on mature pollen grains, which were imaged using a Leica DM5000 B fluorescence microscope as previously described (Yu *et al.*, 2018).

### Evaluation of pollen fertility

To evaluate mature pollen fertility, WT and *des1* mutant anthers were collected and stained with 1% (w/v) iodine–potassium iodide solution (I_2_-KI) and the accumulation of starch in pollen grains was observed using a Leica DM2000 microscope.

### *In vitro* pollen germination assay

An *in vitro* pollen germination assay was performed as described previously (Zhou *et al.*, 2011). Briefly, pollen grains were placed on Brewbaker and Kwack medium (10% sucrose, 200 mg l^–1^ magnesium sulfate, 300 mg l^–1^ calcium nitrate, 100 mg l^–1^ boric acid, and 100 mg l^–1^ potassium nitrate) for 1 h at 25 °C. The pollen grains were observed for germination using a Leica DM2000 light microscope. We defined successful germination as when the elongated length of the pollen tube exceeded the diameter of the pollen grain. The germination rate of the WT and the *des1* mutant was calculated by examining at least 200 pollen grains per genotype.

### Observation of pollen germination on the stigma

Observation of pollen germination on the stigma was performed as described previously (Xu *et al.*, 2017). The pistils of WT and *des1* mutants were fixed in FAA, dehydrated with an ethanol series, incubated in 10 M sodium hydroxide at 56 °C for 8 min, and then stained with 0.1% aniline blue solution. The pistils were imaged using a Zeiss LSM880 laser scanning confocal microscope. Pollen tube growth was defined as when at least one pollen tube in the ovule reached the micropyle.

### Transmission and scanning electron microscopy

Mature anthers from WT and *des1* plants were collected and fixed in 2.5% glutaraldehyde (pH 7.2) for 24 h, fixed in 1% OsO_4_ in phosphate buffer solution, and dehydrated with an ethanol series. Ultra-thin sections were stained with uranyl acetate and aqueous lead citrate solution, and then examined with a Hitachi H-7650 transmission electron microscope. For scanning electron microscopy, the mature anthers and pistils were fixed overnight with 2.5% glutaraldehyde (pH 7.2), rinsed three times using 0.1 M phosphate buffer solution, fixed in 1% OsO_4_ for 1.5 h, and dehydrated through an ethanol series. Subsequently, the samples were subjected to CO_2_ critical point drying, plated with gold by a sputter coater, and observed with a Hitachi TM-1000 scanning electron microscope.

### Map-based cloning

To map the *OsDES1* locus, eight individual plants with abnormal spikelets were chosen from the F_2_ population derived from the cross of 02428 and the *des1* mutant for preliminary mapping using ~200 genome-wide insertion–deletion and simple sequence repeat markers. To fine map the *OsDES1* locus, a total of 393 plants with the mutant phenotype were selected from the F_2_ population and 27 new molecular markers were designed by comparing the nucleotide polymorphisms in the reference sequences between cultivars 9311 and ‘Nipponbare’ (NIP). All primers used for mapping are listed in Supplementary Table S1.

### RNA extraction and quantitative real-time reverse transcription–PCR

Total RNA was extracted from flag leaves at the heading stage and from pistils at different developmental stages using a RNAprep pure Plant kit (Tiangen Biotech Co. Ltd, Beijing, China) following the manufacturer’s instructions. First-strand cDNA was synthesized with a ReverTraAce® qPCR RT Master Mix with a gDNA Remover kit (Toyobo Co. Ltd, Osaka, Japan) using 1.5 μg of RNA. Quantitative real-time reverse transcription–PCR (qRT–PCR) assays were performed with a SYBR premix Ex Taq Kit (Takara Bio Inc., Kusatsu, Shiga, Japan). The relative mRNA levels of the investigated genes were normalized to *Ubiquitin* (*Os03g0234350*) and *Actin* (*Os03g0718100*) by the 2^–ΔΔ^CT calculation method with three replicates, respectively. The primers used for qRT–PCR are shown in Supplementary Table S1.

### Vector construction and plant transformation

For overexpression of *OsDES1*, the WT full-length cDNA was amplified and subcloned into the pCUbi1390 plasmid under the control of the maize *Ubiquitin 1* promoter. The resulting construct was transformed into the *des1* mutant by *Agrobacterium*-mediated transformation. The CRISPR/Cas9 system was used to knock out the *OsDES1* gene as previously reported (Miao *et al.*, 2013; Huang *et al.*, 2017). The vector pBWA(V)HS_cas9i2 containing the target sequence was transformed into NIP callus tissue through *Agrobacterium*-mediated transformation. Individual plants carrying mutations were identified by sequencing before further analysis. For the promoter activity assay of *OsDES1*, a 2444 bp DNA fragment upstream of the *OsDES1* start codon was amplified and ligated into the binary vector pCAMBIA1305 to serve as the *OsDES1* promoter to drive the expression of the *β-glucuronidase* (*GUS*) reporter gene. The construct was transformed into the *japonica* variety NIP. The names and sequences of all primers used for vector construction and sequencing are listed in Supplementary Table S1.

### **β**-**Glucuronidase histochemical staining**

Different tissues from *OsDES1-*promoter*-GUS* transgenic plants of NIP were collected at different developmental stages and stained as previously described (Jefferson, 1989). Images were obtained using a scanner (MRS-9600TFU2L) and a stereomicroscope (Leica MC120HD) with a digital camera.

### RNA *in situ* hybridization

WT spikelets at different developmental stages were fixed overnight in an FAA (RNase-free) fixative solution at 4 °C. After being dehydrated in a graded ethanol series and xylene, pistils were embedded in paraffin. An *OsDES1* cDNA fragment was amplified using primers (listed in Supplementary Table S1) and cloned into the pGEMT Easy vector. The antisense and sense probes were then transcribed *in vitro* using a DIG RNA Labeling Kit (SP6/T7) (Roche) according to the manufacturer’s instructions. RNA hybridization and immunological detection of the hybridized probes were performed as previously described (Kouchi and Hata, 1993).

### Subcellular localization

To determine the subcellular localization of OsDES1, ΔOsDES1 (the mutant OsDES1 protein), and the NEMP domain, the coding sequence (CDS) of *OsDES1*, *ΔOsDES1*, and NEMP was amplified and inserted into the GFP vector pYBA1132. Rice leaf protoplasts were isolated from 10-day-old ZH8015 seedlings. The empty vector (as control) and the recombinant construct plasmids were transfected into protoplasts and incubated for 24 h in the dark (Yoo *et al.*, 2007). The Ghd7-CFP construct was used as a nuclear marker. FM4-64 solution (8.2 μM; Molecular Probes) was added to protoplasts, which were incubated for 15 min to label the membranes and then observed immediately (Wang *et al.*, 2018). The recombinant construct plasmids were co-expressed with Ghd7-CFP in *Nicotiana benthamiana* leaves. After 48 h, the fluorescent signal was detected with a Zeiss LSM710 confocal laser scanning microscope. All primers used are shown in Supplementary Table S1.

### Yeast two-hybrid assay

The CDS of *LOG* was amplified and inserted into the prey vector pPR3-N. The CDS of *OsDES1* was amplified and cloned into the bait vector pBT3-SUC. A yeast two-hybrid assay was performed according to the manufacturer’s instructions (Clontech). The DUALmembrane system was used for conducting the assays. All primers are listed in Supplementary Table S1.

### Split luciferase complementation assay

The CDS of *OsDES1* was cloned into the pCAMBIA-split_nLUC vector, and the CDS of *LOG* was cloned into pCAMBIA-split_cLUC. The constructs were transformed into *Agrobacterium tumefaciens* GV3101 and transiently expressed in *N*. *benthamiana* leaves. The primers used to construct *nLUC-OsDES1* and *cLUC-LOG* are listed in Supplementary Table S1.

### Co-immunoprecipitation assay

The full-length cDNA sequences of *OsDES1* and *LOG* were amplified by PCR and fused with sequences encoding GFP and the Myc tag driven by the *35S* promoter, respectively. The specific primer pairs used to amplify *GFP-OsDES1* and *Myc-LOG* were GFP-OsDES1-F and GFP-OsDES1-R, and Myc-LOG-F and Myc-LOG-R, respectively. Leaves of *N*. *benthamiana* were transfected by injection with *A. tumefaciens* GV3101 containing the *35S:GFP-OsDES1* and *35S:Myc-LOG* constructs as previously described (Voinnet *et al.*, 2003). Total protein was extracted in extraction buffer [20 μg ml^–1^ MG132, 50 mM Tris–HCl, pH 7.5, 150 mM NaCl, 2% Triton X-100, 20% glycerol, 1× complete protease inhibitor cocktail (Roche), and 1 mM EDTA], and incubated with GFP-Trap A agarose beads for 60 min at 4 °C. The beads were rinsed three times with washing buffer [1× complete protease inhibitor cocktail (Roche), 150 mM NaCl, 50 mM Tris–HCl, pH 7.5, and 0.1% Triton X-100]. The immunoprecipitated proteins were separated by 10% SDS-PAGE and analyzed by immunoblot analysis with anti-Myc (LOG) and anti-GFP (OsDES1) antibodies. All primers used are shown in Supplementary Table S1.

## Results

### Identification of a low seed-setting rate rice mutant

**Fig. 1.**
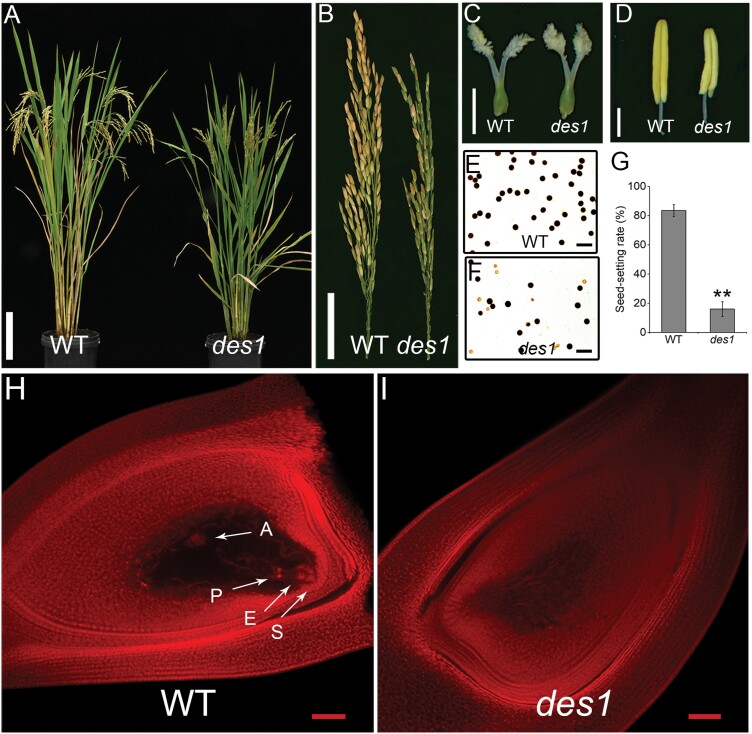
Phenotypic characterization of *des1* rice. (A) Comparison of WT and *des1* plants at maturity. Scale bar=20 cm. (B) Mature panicles of WT and *des1* plants. Scale bar=5 cm. (C) Pistils of WT and *des1* at maturity. Scale bar=0.125 cm. (D) Anthers of WT and *des1*. Scale bar=1.25 mm. (E, F) I_2_-KI staining of pollen grains in WT (E) and *des1* (F). Scale bars=25 μm. (G) Statistical analysis of the seed-setting rates in WT and *des1* plants. Data are means ±SD (*n*=8 plants). Asterisks indicate significant differences (**P<0.01; Student’s *t*-test). (H, I) Microscopic observations of mature embryo sacs in WT (H) and *des1* (I). Arrows indicate the eight-cell components in the embryo sac: A, antipodal cell; E, egg cell; P, polar nucleus; S, synergid cell. Scale bars=50 μm.

In an effort to characterize rice sterility phenotypes, we identified a low-seed-setting rice mutant, named *defective embryo sac 1* (*des1*), from a ^60^Co-γ-irradiated library of the *indica* rice cv. ZH8015. Compared with the WT, *des1* plants were slightly shorter in height and showed earlier heading but were otherwise normal in vegetative development (Supplementary Fig. S1A). The seed-setting rate of the WT was ~84%, whereas *des1* showed a much lower seed-setting rate (~16%) under normal field conditions (Fig. 1A, B, G). In addition, *des1* plants produced shorter brown panicles (Fig. 1B). However, no clear difference in pistil morphology was observed in *des1* compared with the WT (Fig. 1C; Supplementary Fig. S2A, B). The WT embryo sacs contained egg cells, synergids, polar nuclei, and antipodal cells, whereas *des1* embryo sacs degenerated and did not have these characteristic structures (Fig. 1H, I). In addition, the most obvious differences in the male reproductive organs between *des1* and the WT were the number of stamens and pollen fertility. Seven different types of stamens were detected in *des1* spikelets, and the numbers of stamens differed in each spikelet, whereas only six stamens were typically found in WT spikelets (Supplementary Fig. S1C, E). The stamen lengths were also shorter, whereas the development of other floral organs appeared normal, in *des1* (Fig. 1D; Supplementary Fig. S2C, D). At anthesis, spikelet development in *des1* was similar to that of the WT (Supplementary Fig. S1B). Pollen viability was approximately 98% in the WT but only 64% in *des1* (Fig. 1E, F; Supplementary Fig. S1D). To determine the causes underlying the reduced fertility observed in *des1*, we performed reciprocal cross experiments. The results showed that the seed-setting rate of WT♀×WT♂ and *des1*♀×*des1*♂ crosses with full pollination were 74.4% and 29.3%, respectively. When the WT was pollinated with *des1* pollen, the hybrid seed-setting rate was 57.3%. However, when *des1* was used as the pollen recipient, the hybrid seed-setting rate was 35.4% (Supplementary Fig. S3). This finding suggests that the low seed-setting rate of *des1* is mainly due to a maternal defect. The F_2_ population showed an approximate 3:1 segregation ratio of normal (108) and low (28) seed-setting (χ^2^=0.235<χ^2 0.05^=3.84, χ^2^ test). These results indicate that female sterility in *des1* was inherited as a single recessive mutation.

### Disrupted embryo sac development in *des1*

**Fig. 2.**
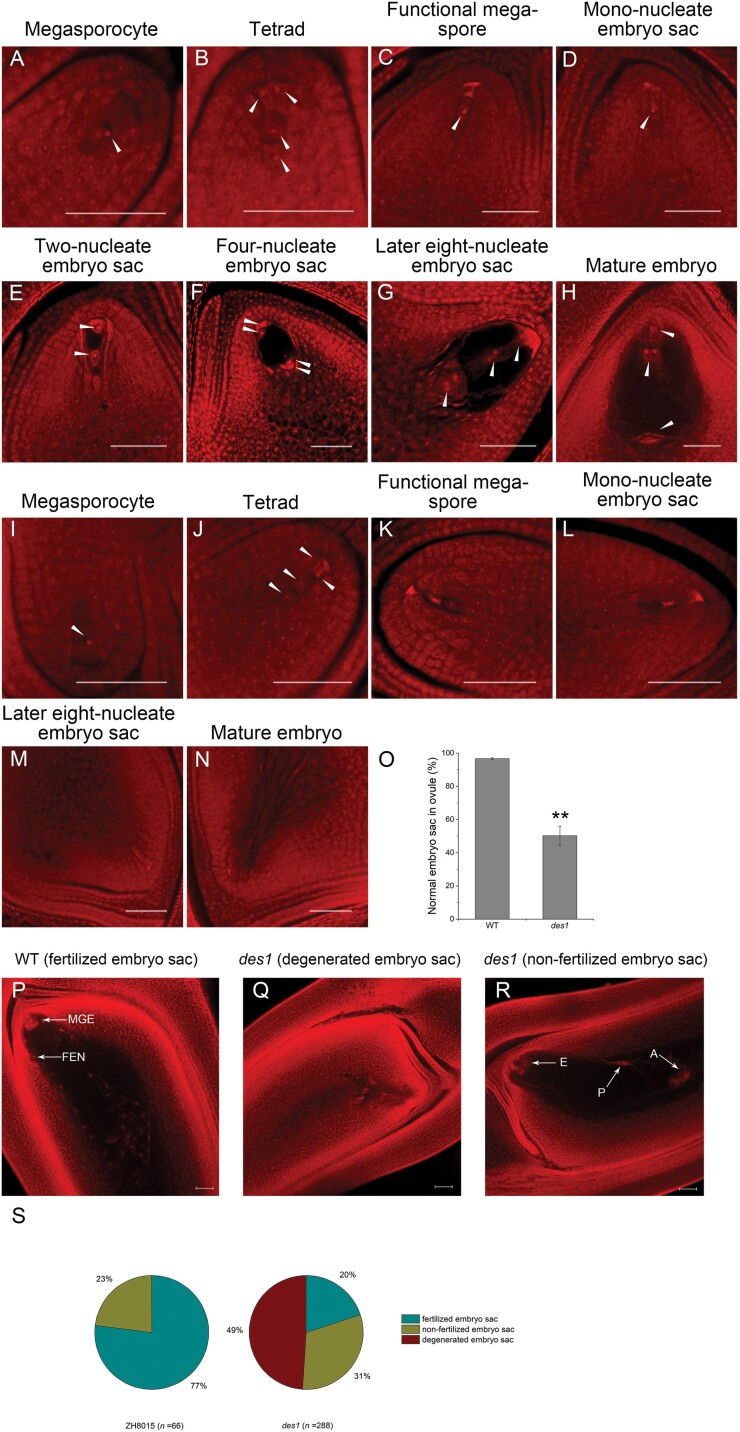
Development of the embryo sac in WT and *des1* rice. (A–N) Different developmental stages of the embryo sac are shown in WT (A–H) and *des1* (I–N). Arrows indicate nuclei during megasporogenesis and megagametogenesis. Scale bars=50 μm. (O) Statistical analysis of normal mature embryo sac formation in WT and *des1*. Data are means ±SD (*n*=3) (ovule number: WT 271, *des1* 607). (P–R) Morphology of embryo sacs 24 h after pollination in WT and *des1*: (P) Normal WT embryo sac with a multicellular globular embryo (MGE) and free endosperm nuclei (FEN); (Q, R) Abnormal *des1* embryo sacs displaying a degenerated embryo sac (Q) and non-fertilized embryo sac (R). A, Antipodal cell; E, egg cell; P, polar nucleus. Scale bars=50 μm. (S) Statistical analysis of embryo sac morphology 24 h after pollination in WT (left) and *des1* (right).

To uncover the cytological basis of female sterility, we compared the formation and development of the embryo sac of the WT and *des1* using whole-mount stain-clearing laser scanning confocal microscopy. In the WT, megasporocytes undergo two meiotic divisions to produce a tetrad of megaspores (Fig. 2A, B). Subsequently, three megaspores situated at the micropylar end degenerate and the remaining megaspore remains functional and develops further by enlargement at the functional megaspore formation stage (Fig. 2C). The mono-nucleate embryo sac undergoes three rounds of mitotic division to form a two-nucleate, four-nucleate, and eight-nucleate embryo sac (Fig. 2D–G). Finally, the mature embryo sac forms, with polar nuclei, the egg cell, synergids, and antipodals (Fig. 2H). The megasporocyte and tetrads form normally in *des1* (Fig. 2I, J), but some embryo sacs exhibit defects at the functional megaspore formation stage. The megaspore near the chalaza in *des1* did not continue to grow into a functional megaspore. Instead, it gradually degenerated along with the other three megaspores near the micropyle (Fig. 2K). The aberrant nuclei then also began to degenerate along with the embryo sac in *des1*. Subsequently, only degenerated footprints of nuclei were observable, and these remained visible for a long time until embryo sac maturity. During the final developmental stage, the embryo sac ultimately developed into undifferentiated tissue, likely due to impaired mitotic processes (Fig. 2L–N). Additionally, ~97% of mature embryo sacs in the WT successfully formed eight-nucleate embryo sacs, whereas in *des1*, only ~50% of mature embryo sacs fully matured to this stage and the remaining embryo sacs degenerated (Fig. 2O). Furthermore, we observed the embryo sac in the WT and *des1* by microscopic examination of paraffin sections. Consistent with the above results, *des1* showed normal formation of the megasporocyte and tetrads, but no functional megaspores and undifferentiated embryo sacs were detected at maturity (Supplementary Fig. S4).

We also observed embryo sac development during the 24 h following pollination in both the WT and *des1*. Both multicellular globular embryos and free endosperm nuclei were observed in the WT, whereas fertilization did not occur in many of the *des1* embryo sacs and most embryo sacs had degenerated or were not fertilized. Approximately 20% of embryo sacs developed normally and became fertilized in *des1*, whereas the proportion of successfully fertilized embryo sacs was 77% in the WT (Fig. 2P–S). This finding indicates that a reduced capacity for fertilization contributed to the low seed-setting rate observed in *des1* (Fig. 1G). Together with our phenotypic observations, this suggests that the mutation in *OsDES1* causes multiple defects in embryo sac formation and fertilization. In addition, we also observed the embryo sacs in *des1*/ZH8015 F_1_ plants. The results showed that normal embryo sacs were detected in 96.1% (*n*=179) of ovules and F_1_ plants were fertile (Supplementary Fig. S5), consistent with a *des1* having a sporophytic effect on embryo sac development.

### Abnormal stamen development and pollen tube growth in *des1*

We observed anther and pollen development in the WT and *des1* and found that the number of aborted pollen grains identified by I_2_-KI staining was lower in the WT than in *des1* (Fig. 1E, F; Supplementary Fig. S1D). We also performed *in vitro* pollen germination assays and found that, compared with WT pollen (~92% viable), only ~61% of *des1* pollen grains successfully germinated, consistent with the results of I_2_-KI staining (Supplementary Fig. S6). Subsequently, we observed pollen germination on the stigma and pollen tube growth in the ovary in both the WT and *des1*. In the WT, 84% of pollen tubes in the ovule were able to grow and reach the micropyle, compared with 80% in *des1* (Supplementary Fig. S7; Supplementary Table S2). To further characterize the differences in pollen development in the WT and *des1*, we examined microspores using acetocarmine and DAPI staining. Observations of microspores showed no clear differences between the WT and *des1* until the mono-nucleate stage (Supplementary Fig. S8A–E, H–L). In *des1*, only a proportion of the pollen grains were able to undergo the first and second mitoses normally, while the remaining pollen grains maintained a single brightly staining nucleus at the bicellular stage (Supplementary Fig. S8F, M). The abnormal pollen grains in *des1* also formed irregular shapes, and became abortive at maturity (Supplementary Fig. S8G, N–P).

No obvious differences between the WT and *des1* were observed in semi-thin sections until the early microspore stage (Supplementary Fig. S9A–I, M). In *des1*, the pollen underwent vacuolization and the tapetum appeared much thicker and did not undergo complete degeneration (Supplementary Fig. S9J, K, N, O). Only a proportion of *des1* microspores ultimately exhibited normal development, and the remaining microspores degenerated (Supplementary Fig. S9L, P). We also compared mature anther and pollen grain morphology between *des1* and the WT by scanning and transmission electron microscopy. Compared with the WT, the *des1* anther epidermis was more compact (Supplementary Fig. S2E, F) and the number of Ubisch bodies was higher (Supplementary Fig. S2G, H). A subset of *des1* pollen grains exhibited a normal plump appearance (Supplementary Fig. S2I, J, L, M), while the remaining pollen grains were shrunken and had abnormal annular protrusions (Supplementary Fig. S2K, N). The pollen grains in the WT had a plump morphology and their internal structure was normal in appearance and contained starch granules (Supplementary Fig. S10A, B). By contrast, the shrunken pollen grains in *des1* contained no starch granules (Supplementary Fig. S10D, E). The tectum and foot layer of *des1* pollen grains were thicker than those in the WT, and the columella was degraded in *des1* (Supplementary Fig. S10E) In addition, the tapetum in *des1* was not completely degraded at maturity, as it was in the WT (Supplementary Fig. S10C, F). These results indicate that the *des1* mutation affects anther and pollen development.

### Map-based cloning of *OsDES1*

**Fig. 3.**
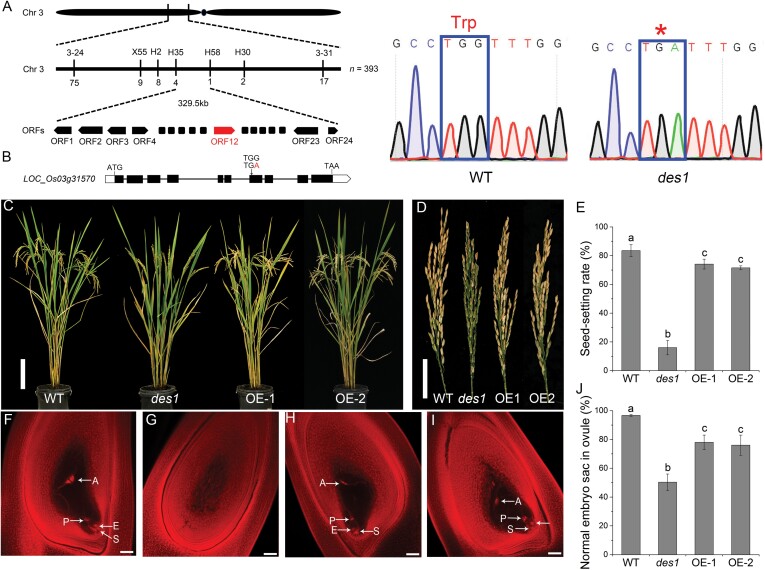
Map-based cloning of *OsDES1*. (A) Mapping of the *OsDES1* locus. The molecular marker loci and numbers of recombinant plants are shown above and below the line, respectively. The candidate gene *OsDES1*/*LOC_Os03g31570* is shown in red. ORF, Open reading frame. (B) Schematic representation of the *OsDES1* gene. White boxes, black boxes, and black lines indicate untranslated regions, exons, and introns, respectively. The single-base substitution of A for G in the seventh exon is shown. (C) Morphology of mature WT, *des1*, and *OsDES1*-overexpressing (OE-1 and OE-2) plants. Scale bar=20 cm. (D) Mature panicles of WT, *des1*, and OE plants. Scale bar=5 cm. (E) Seed-setting rates in WT, *des1*, and *OsDES1*-overexpressing plants. Data are means ±SD (*n*=8 plants). Different letters indicate significant differences (*P*<0.05; Duncan’s test). (F–I) Microscopic observations of mature embryo sacs in WT (F), *des1* (G), and OE lines (H, I). A, antipodal cell; E, egg cell; P, polar nucleus; S, synergid cell. Scale bars=50 μm. (J) Statistical analysis of normal mature embryo sac formation in WT, *des1*, and OE plants. Data are means ±SD (*n*=3) (ovule number: WT 177; *des1* 162; OE 267). Different letters indicate significant differences (*P*<0.05; Duncan’s test).

To identify the causal gene responsible for female sterility, we first crossed the *des1* mutant with the *japonica* rice cultivar 02428 to generate an F_2_ mapping population. Using 393 recessive plants from the F_2_ population, we mapped *OsDES1* to a 329.5 kb region on chromosome 3 located between marker loci H35 and H58, where a total of 24 open reading frames were predicted (Fig. 3A). Genomic sequence analysis revealed that *LOC_Os03g31570* carried a nonsense mutation in the seventh exon in *des1* (Fig. 3B). *LOC_Os03g31570* was predicted to contain 10 exons and nine introns and encodes a 485 amino acid protein with a putative NEMP domain at amino acid residues 157–403 (https://www.ncbi.nlm.nih.gov/Structure/cdd/wrpsb.cgi). The mutation in *des1* led to a truncated amino acid sequence lacking the NEMP domain.

**Fig. 4.**
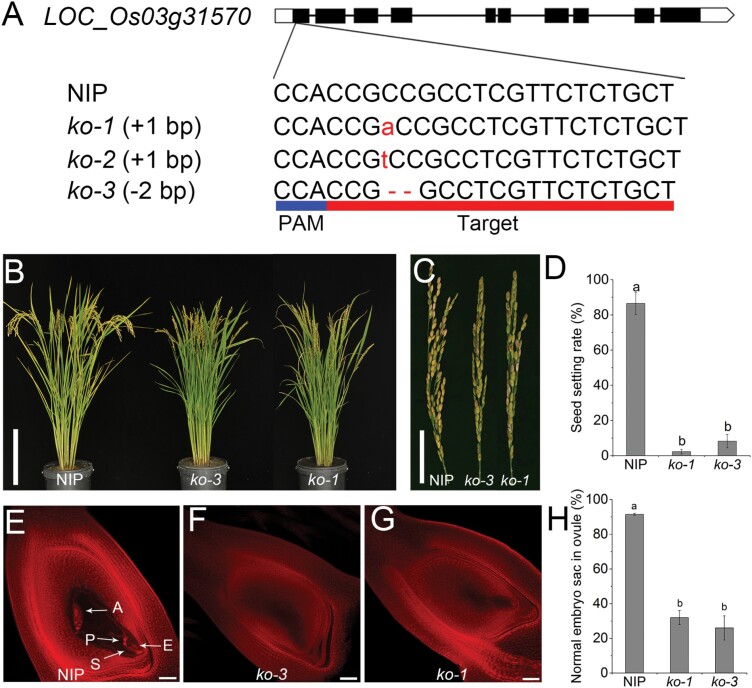
CRISPR/Cas9 mutation of *OsDES1*. (A) Three types of mutations detected in the target site in the knockout (*ko*) lines. (B) Comparison of mature plants of NIP and the *ko* lines. Scale bar=20 cm. (C) Panicles of NIP and *ko* plants at maturity. Scale bar=5cm. (D) Seed-setting rate of NIP and *ko* plants. Data are means ±SD (*n*=10 plants). Different letters indicate significant differences (*P*<0.05; Duncan’s test). (E–G) Microscopic observations of mature embryo sacs in NIP (E) and *ko* (F, G) plants. A, antipodal cell; E, egg cell; P, polar nucleus; S, synergid cell. Scale bars=50 μm. (H) Statistical analysis of the numbers of normal mature embryo sacs in NIP and *ko* plants. Data are means ±SD (*n*=3) (ovule number: NIP 140; *ko* 292). Different letters indicate significant differences (*P*<0.05; Duncan’s test).

To verify whether the mutation in *OsDES1* is responsible for the *des1* mutant phenotype, we overexpressed *OsDES1* under the control of the maize *Ubiquitin1* promoter in the *des1* background. As expected, the seed-setting rate in *OsDES1*-overexpressing plants was substantially increased compared with *des1* (Fig. 3C–E). In addition, developmental defects observed in the spikelet, mature embryo sac, anther, and pollen grain were largely rescued in the transgenic plants grown under natural field conditions (Fig. 3F–J; Supplementary Fig. S11A–F). To further confirm that the mutation in *OsDES1* is responsible for the mutant phenotype, we used CRISPR/Cas9 to generate five knockout mutant lines in the NIP background (Fig. 4A). Sequencing analysis revealed that these lines harbored three different types of independent homozygous mutations. All of these mutations resulted in predicted translational frame shifts (Fig. 4A). As expected, all knockout transgenic plants had the same phenotype as the *des1* mutant, with the characteristic decreased seed-setting rate. The seed-setting rate of the WT (NIP) was ~87%, while both the *ko-3* mutant (~8%) and *ko-1* mutant (~2%) showed a significantly lower seed-setting rate (Fig. 4B–D). Similarly, ~92% of NIP mature embryo sacs successfully formed eight nuclei, whereas in the *ko-3* and *ko-1* lines only ~26% and ~32%, respectively, of mature embryo sacs had a normal appearance, and the rest developed into undifferentiated tissue (Fig. 4E–H). In addition, smaller brown panicles, shorter anthers, and abortive pollen grains were observed in the homozygous knockout lines (Supplementary Fig. S12). Together, these results confirmed that *LOC_Os03g31570* corresponds to *OsDES1*, and mutation of this gene resulted in a low seed-setting rate.

### Subcellular localization of OsDES1 protein and expression pattern of the *OsDES1* gene

**Fig. 5.**
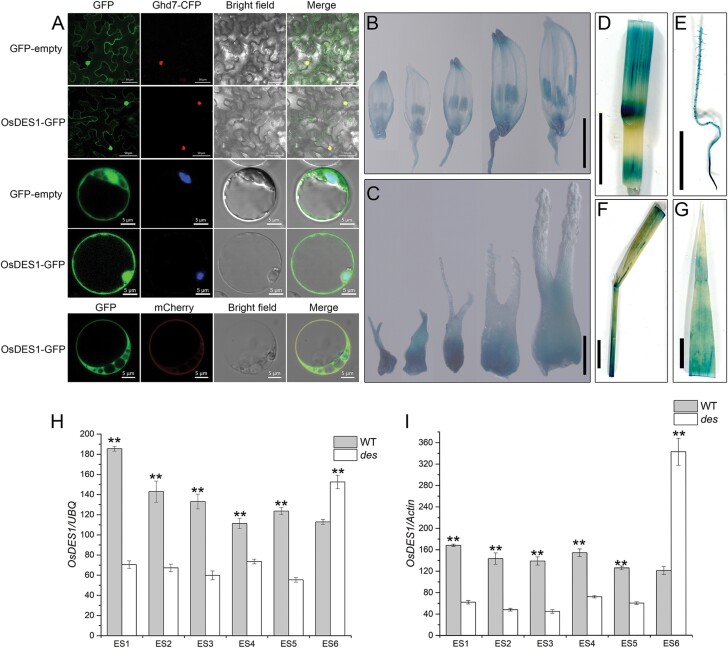
Subcellular localization of OsDES1 and expression analysis of the *OsDES1* gene in rice tissues. (A) Subcellular localization of OsDES1-GFP fusion protein in *N. benthamiana* leaf epidermal cells (top two rows) and rice leaf protoplasts (bottom three rows). Ghd7-CFP fusion protein was used as a nuclear marker. The plasma membrane was stained with FM4-64. (B–G) GUS staining of various tissues of pro*OsDES1*-GUS transgenic plants: spikelets (B) and pistils (C) at different developmental stages, stem (D), primary root (E), leaf sheath (F), and leaf (G). The lengths of the spikelets from left to right in (B) and (C) are 2.5–3.0 mm, 3.0–3.4 mm, 3.4–4.5 mm, 4.5–5.6 mm, and 5.6–6.2 mm. Scale bars=50 μm and 5 μm in (A), 2 mm in (B), 200 μm in (C), and 1 cm in (D–G). (H, I) Expression levels of *OsDES1* in WT and *des1* during embryo sac development. ES1, spikelet lengths 5–5.9 mm; ES2, spikelet lengths 6–6.9 mm; ES3, spikelet lengths 7–7.9 mm; ES4, spikelet lengths 8–9.9 mm; ES5, spikelet lengths >10 mm; ES6, mature spikelets. The *UBQ* and *Actin* genes were used as internal controls for the data in (H) and (I), respectively. Data are the means ±SD of three independent biological replicates. Asterisks indicate significant differences (**P<0.01; Student’s *t*-test).

*OsDES1* encodes a putative NEMP containing seven putative transmembrane regions. To determine the subcellular localization of OsDES1, we fused the full-length CDS of *OsDES1* to the N-terminus of GFP driven by the CaMV35S promoter. In rice leaf protoplasts, GFP signals were clearly detected in the nuclear membrane, nucleus, plasma membrane, and cytoplasm (Fig. 5A). Notably, the ΔOsDES1 and NEMP domain showed a similar subcellular localization pattern to that of OsDES1 in rice leaf protoplasts (Supplementary Fig. S13). These results indicated that the mutation of *OsDES1* did not change the ­subcellular localization of its protein product and the NEMP domain may play a crucial role for the function of OsDES1. The results of these experiments were further confirmed in *N. benthamiana* leaves, which displayed similar results to the rice leaf protoplasts. 

We then examined whether *OsDES1* is transcriptionally expressed in specific tissues or during specific developmental stages in rice. To this end, we generated transgenic plants expressing the *OsDES1*_*pro*_*::GUS* reporter construct. Our results showed that *OsDES1* is expressed in a range of rice tissues. Notably, the expression was strong in anthers (Fig. 5B) and pistils (Fig. 5C) from meiosis to maturity, whereas expression was found to be relatively weak in the culms (Fig. 5D), young roots (Fig. 5E), leaf sheaths (Fig. 5F), and leaves (Fig. 5G). To further elucidate the function of OsDES1 during embryo sac development, we analyzed the expression of *OsDES1* in pistils of the WT and *des1* using qRT–PCR at different developmental stages. In the megasporocyte (ES1), dyad (ES2), tetrad (ES3), functional megaspore formation (ES4), and mitosis (ES5) stages, the expression of *OsDES1* in the WT was significantly greater than that in *des1*; however, the expression of *OsDES1* was obviously lower in the WT at the mature stage (ES6) (Fig. 5H, I), which was consistent with the results of GUS staining (Fig. 5C). 

To further elucidate the temporal and spatial expression patterns of *OsDES1*, we performed RNA *in situ* hybridization with WT pistil sections. The hybridization signals were detected in whole ovules, including embryo sacs, inner integuments, and outer integuments. As expected, strong signals were observed in the ovule at the megasporocyte stage, tetrad, functional megaspore formation stage, and mature embryo sac stage (Supplementary Fig. S14). The results of *in situ* hybridization are consistent with those of GUS staining and qRT–PCR, indicating that OsDES1 functions in ovules in reproductive development.

### OsDES1 interacts with LOG

**Fig. 6.**
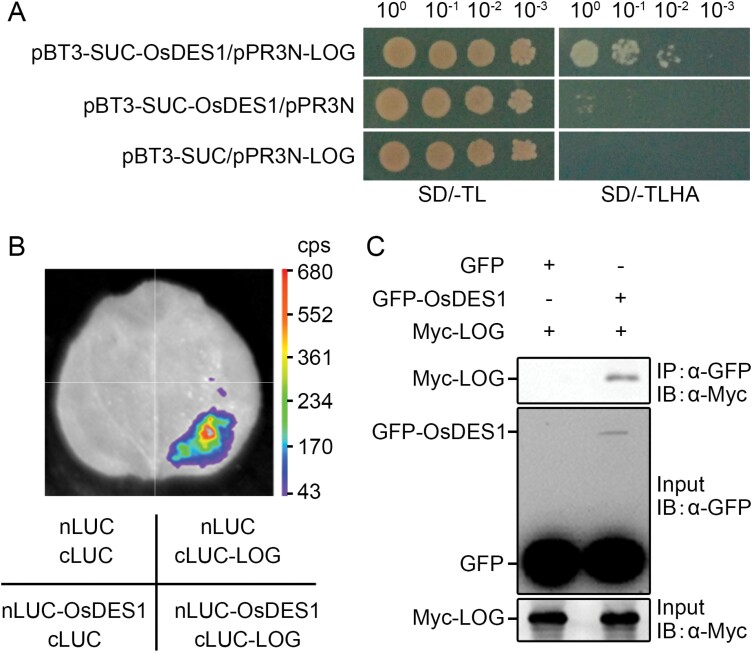
Physical interaction of the OsDES1 and LOG proteins. (A) Yeast two-hybrid assay to detect the interaction between OsDES1 and LOG. The pPR3N/pBT3-SUC pair was used as the negative control. SD/-TL, Synthetic dropout medium lacking Trp and Leu; SD/-TLHA, synthetic dropout medium lacking Trp, Leu, His, and Ade. (B) Split luciferase complementation assay showing the interaction between OsDES1 and LOG in *N. benthamiana.* nLUC-OsDES1 and cLUC-LOG were co-expressed in *N. benthamiana* leaves. Luciferase activity was tested 24 h after infiltration. nLUC and cLUC were used as negative controls. (C) Co-immunoprecipitation assay confirming the interaction between OsDES1 and LOG. *35S:GFP-OsDES1* and *35S:Myc-LOG* constructs were co-expressed in *N. benthamiana.* Proteins were immunoprecipitated (IP) using GFP beads and analyzed by immunoblotting (IB) with anti-Myc and anti-GFP antibodies.

Previous studies showed that the mutation of *LOG*, a gene encoding a cytokinin-activating enzyme, resulted in similar phenotypic defects to *des1* (Kurakawa *et al.*, 2007; Yamaki *et al.*, 2011). To identify potential interaction partners involved in OsDES1-mediated female organ development, we conducted yeast two-hybrid screening for OsDES1-interacting proteins. These assays revealed that LOG interacts with OsDES1 in yeast cells *in vitro* (Fig. 6A). To further verify this interaction, we carried out split luciferase complementation assays. Leaves of *N. benthamiana* co-transfected with *nLUC-OsDES1* and *cLUC-LOG* constructs showed significant luciferase activity, whereas the negative control produced no luciferase signal, indicating that OsDES1 interacts with LOG *in vivo* (Fig. 6B). To further confirm the association between OsDES1 and LOG *in planta*, we used co-immunoprecipitation analysis to detect their interactions *in vivo*. We transiently co-expressed *35S:GFP-OsDES1* and *35S:Myc-LOG* in *N*. *benthamiana*, with *35S:GFP* and *35S:Myc-LOG* serving as negative controls. As shown in Fig. 6C, we found that Myc-LOG interacts with GFP-OsDES1 but not with GFP *in vivo*. Thus, these results demonstrate that OsDES1 interacts with LOG both *in vivo* and *in vitro*. 

*LOG* encodes a cytokinin-activating enzyme that acts in bioactive cytokinin synthesis (Kurakawa *et al.*, 2007). Hence, we examined endogenous cytokinin levels in pistils of the WT and *des1* at maturity by HPLC, and found that the levels of several cytokinins were significantly higher in the pistils of *des1* compared with those of the WT (Supplementary Fig. S15A). We also assessed the expression levels of genes involved in the cytokinin signaling pathway, including cytokinin-response histidine protein kinases (*OsHKs*), histidine phosphotransfer proteins (*OsHPs*), and cytokinin response regulators (*OsRRs*). *OsRR1*, *OsRR4*, *OsRR9*, *OsRR10*, *OsRR16*, *OsRR19*, *OsRR20*, *OsHK3*, *OsHK4*, *OsHP1*, *OsHP2, OsHP3*, *OsHP4*, and *OsHP5* were up-regulated in *des1* (Supplementary Fig. S15D–G). The expression levels of *LOG* were significantly reduced in the pistils of *des1* compared with the WT (Supplementary Fig. S15B, C). The expression levels of *OsDES1* in *des1* were significantly increased at the mature embryo stage, and were significantly higher than in the WT (Fig. 5H, I). Hence, we speculated that *OsDES1* is involved in the regulation of cytokinin.

## Discussion

The seed-setting rate is a major agronomic character that directly contributes to grain yield. Defective female reproductive organs lead to reduced fertility, which is one of the most common reasons for a reduction in the seed-setting rate in rice (Ren *et al.*, 2019; Xu *et al.*, 2020). In this study, we used map-based cloning to isolate a *des1* rice mutant that exhibited an extremely low seed-setting rate. Cytological and genetic studies suggested that the low seed set in *des1* is mainly caused by abnormal embryo sac development (Figs 2O, 3E, 4D). Similar cases have been reported previously. *OsMLH3*, which encodes a MutL-homolog 3 protein in rice, positively controls the panicle seed-setting rate by regulating embryo sac development (Mao *et al.*, 2021). A reduced seed-setting rate can also result from different mechanisms. The *female sterile variation 1* (*fsv1*) mutant shows low seed set, which is attributed to non-functional embryo sacs, while the low seed-setting rate of *des1* is due to degeneration of the embryo sac and a reduced capacity for fertilization.

In *OsDES1-*overexpressing plants, the seed-setting rate and fertility were substantially increased compared with *des1* (Fig. 3E, J; Supplementary Fig. S11F). However, the overexpression construct was driven by the maize *Ubiquitin1* promoter instead of the endogenous promoter, and the relative transcription levels of *OsDES1* were significantly higher than in the WT (Supplementary Fig. S11G, H), which may have interfered with seed setting, thus preventing a complete rescue. In our study, seed set was lower than embryo sac fertility in *des1* (Figs 1G, 2O). When *des1* was used as the maternal parent, varying degrees of reduced seed-setting rates (ranging from 29.3% to 35.4%) were identified. This finding suggests that low seed set in *des1* is mainly due to a maternal effect. In addition, when the WT was used as the maternal parent in a cross with *des1*, we found a lower seed-setting rate than with WT self-crosses (Supplementary Fig. S3). These data suggest that fertilization is abnormal in both *des1* and reciprocal crosses. Thus, we can hypothesize that there are other factors influencing seed setting in the *des1* mutant besides embryo sac fertility.

Fertilization is a complex and robust process that is the core process in the reproductive development of angiosperms. The key to the success of fertilization is that two sperm cells can individually migrate to the egg cell and polar nuclei for karyogamy, ultimately forming viable seeds (Russell, 1996; Yadegari and Drews, 2004; Skinner and Sundaresan, 2018). Reduction in fertilization ability is another cause of the low seed-setting rate in *des1*. Approximately 51% of the embryo sacs were normal in *des1*. However, only ~20% of the normal embryo sacs in *des1* were fertilized, compared with 77% in the WT. These findings suggest that both the egg cell and the polar nuclei do not undergo successful fertilization in some *des1* normal embryo sacs at 24 h post fertilization and, as a result, seed set is reduced (Fig. 2P–S). Previous studies have shown that the fertilization process is affected by many factors, such as gametogenesis, pollen tube growth in the ovule, pollen tube reception, sperm cell release, and the recognition, activation, and fusion of male and female gametes (Berger, 2011; Dresselhaus *et al.*, 2016; Sankaranarayanan and Higashiyama, 2018; Manrique *et al.*, 2019; Sun *et al.*, 2021). We observed pollen germination on the stigma and pollen tube growth in the ovule at 2 h after pollination in the WT and *des1*. The pollen tube could arrive at the micropyle at 2 h after pollination in *des1*, as in the WT (Supplementary Fig. S7; Supplementary Table S2). We speculate that there may be one or more points that could fail after the pollen tubes reach the micropyle, for example, in sperm cell delivery or the recognition, activation, or fusion of the gametes. Further studies are required to address this issue.

Through cytological and genetic studies, we found that sterility in *des1* is mainly caused by abnormal development of the embryo sacs. Ultimately, *des1* embryo sacs failed to divide or differentiate correctly, resulting in plant sterility (Fig 2A–N). Furthermore, reciprocal cross experiments also indicated that there are functional defects in the female reproductive organ of *des1* (Supplementary Fig. S3). The expression of *OsDES1* in the pistil was consistent with its functions (Fig. 5C, H, I; Supplementary Fig. S14). Therefore, *OsDES1* plays a vital role in the regulation of embryo sac development in rice. Several studies have shown that defects in pollen and embryo sac development lead to sterility in *indica–japonica* hybrids (Song *et al.*, 2005; Long *et al.*, 2008; Zeng *et al.*, 2009; Yang *et al.*, 2012). Embryo sac fertility and pollen fertility are considered to be the most critical factors affecting spikelet fertility (Song *et al.*, 2005; Zeng *et al.*, 2009). The egg cell and central cell in the embryo sac develop into the embryo and endosperm by fertilization. Studies of the female gametophyte in flowering plants promote understanding of the molecular mechanism for cell specification, cell–cell interaction, and programmed cell death (Heydlauff and Groß-Hardt, 2014; Tekleyohans *et al.*, 2017).

The process of embryo sac development involves the development of the archesporial cell and megaspore mother cell, functional megaspore formation, and gamete cell differentiation. Functional megaspore formation, which is known as the origin of the gametophytic lineage, is essential for embryo sac development (Demesa-Arévalo and Vielle-Calzada, 2013). Disruption of functional megaspore formation may cause female sterility. The *osrpa1a*, *Osmsh4*, and *fsv1* mutants show defects in the megaspore at the tetrad stage, which lead to failures in functional megaspore formation (Chang *et al.*, 2009; Wang *et al.*, 2016; Mao *et al.*, 2021). Cytological observation has shown that, unlike in the *osrpa1a*, *Osmsh4*, and *fsv1* mutants, the megasporocyte can give rise to a tetrad megaspore in *des1*. However, the megaspore at the chalaza degenerated, together with the other three degenerating megaspores nearer the micropylar end, at the functional megaspore formation stage. Ultimately, the selected megaspore was unable to develop into a functional megaspore, which subsequently led to the formation of an undifferentiated embryo sac and female sterility (Fig. 2I–N). Intriguingly, female sterility in *des1* is similar to that observed in plants in which *DEFECT IN EARLY EMBRYO SAC1* (*OsDEES1*) has been silenced by RNAi, since both show normal tetrad megaspore and abnormal embryo sac formation (Wang *et al.*, 2012). However, *des1* shows defects during functional megaspore formation, whereas the *OsDEES1* RNAi plants exhibit normal functional megaspore formation and severe defects in mitosis.

Previous results indicated that *LOG* regulates the development of the pistil and ovule. LOG activates cytokinin by catalyzing the conversion of inactive cytokinin species to active forms (Kurakawa *et al.*, 2007; Yamaki *et al.*, 2011). Therefore, the *log-3* mutant is mainly considered to be a female-sterile mutant (Yamaki *et al.*, 2011). Our results revealed a physical interaction between OsDES1 and LOG *in vitro* and *in vivo* (Fig. 6). Furthermore, LOG functions in the cytosol (Kurakawa *et al.*, 2007), and OsDES1 was also detected in the cytoplasm (Fig. 5A). In addition, we found that the *des1* and *log* mutants showed similar phenotypes in female gamete development. Therefore, we speculated that OsDES1 and LOG have a complex interaction in the regulation of cytokinin synthesis, and then both of them participate in the whole developmental process of embryo sacs from initiation to maturity. OsDES1 was also found to be localized to the nuclear membrane and plasma membrane in rice leaf protoplasts (Fig. 5A). This observation implies that OsDES1 might function in other biological pathways in rice reproductive development. Cytokinin is involved in diverse physiological functions, including cell proliferation, differentiation, shoot apical meristem function, seed germination, delayed leaf senescence, and plant immunity (Argueso *et al.*, 2012; Hwang *et al.*, 2012). In Arabidopsis, cytokinin signaling at the chalazal end of the developing embryo sac appears to be necessary for the selection of the functional megaspore. When cytokinin signaling in the sporophyte is disturbed, it leads to abnormalities in the functional megaspore. It has been shown that there is an uneven distribution of cytokinin signaling and biosynthesis in the ovule, particularly in the chalaza during megasporogenesis (Cheng *et al.*, 2013). In maize, cytokinin signaling is not detected in the embryo sac, but in the outer periphery of the antipodal cells (Chettoor and Evans, 2015). Our results showed that the levels of several cytokinins were significantly higher in the mature pistils of *des1* compared with the WT (Supplementary Fig. S15A). Hence, we speculate that cytokinin may participate in the regulation of female reproductive organ development. Future studies are needed to determine how cytokinin regulates female reproductive organ development in rice.

*OsDES1* encodes a putative NEMP domain-containing protein. The nuclear envelope not only protects the genome from detrimental agents but also governs genome organization (Yang *et al.*, 2017). The NEMP family includes a group of nuclear envelope integral membrane proteins in animals and plants. Nup154 is similar to known nucleoporins, and it is localized both at the nuclear envelope and in the nuclear interior, which further confirms its numerous roles in nuclear functions. In the ovary, *Nup154* is required for egg chamber development and oocyte growth (Gigliotti *et al.*, 1998). In Arabidopsis, loss of function of nucleoporin 1 (NUP1) causes defects in both female and male gametogenesis (Bao *et al.*, 2019). Recently, two new homologs of rice OsDES1 (PNET1 and PNET2) have been characterized in Arabidopsis. PLANT NUCLEAR ENVELOPE TRANSMEMBRANE (PNET1) interacts with the nuclear pore complex outer ring complex nucleoporin Nup160 and is required for embryo development. *nup160/pnet1* double mutants are embryo lethal and show undeveloped ovules and a reduction in seed set. However, the single *pnet1* and *nup160* mutants show normal development and seed set (Tang *et al.*, 2020). PNET2 plays essential roles in establishing chromatin architecture and transcription programming. Both *pnet2* single and triple mutants showed distinct defects in plant growth and development (Tang *et al.*, 2022). In our study, *des1* and the CRISPR/Cas9-based knockout lines had a low seed-setting rate. In addition, OsDES1 localized to the nuclear membrane, consistent with the previous localization of PNET1 and PNET2. The same-origin gene mutation causes diverse phenotypes. These results suggest that the NEMP protein OsDES1 in rice regulates embryo sac development and seed set. Our study provides new insight into the function of a NEMP protein in regulating reproductive development in a monocot, unlike other known and characterized NEMP proteins. In summary, our findings elucidate the essential regulatory mechanism of OsDES1 in embryo sac and pollen development, which may contribute to applications in rice production and the improvement of rice yield.

## Supplementary data

The following supplementary data are available at *JXB* online.

Fig. S1. Phenotypic analysis in WT and *des1*.

Fig. S2. Scanning electron microscopy observation of the mature pistils, anthers, and pollen grains in WT and *des1*.

Fig. S3. Statistical data of the seed-setting rate of the reciprocal crosses.

Fig. S4. Paraffin section analysis of embryo sac development in WT and *des1*.

Fig. S5. Microscopic observations of mature embryo sacs in ZH8015 and F_1_ plants.

Fig. S6. *In vitro* pollen germination assay.

Fig. S7. Pollen germination on the stigma and pollen tube growth in WT and *des1*.

Fig. S8. Male gametogenesis in WT and *des1* shown by acetocarmine and DAPI staining.

Fig. S9. Transverse sections of WT and *des1* anthers at various developmental stages.

Fig. S10. Transmission electron microscopy observations of mature anthers in WT and *des1*.

Fig. S11. Anthers and pollen grains of WT, *des1*, and *OsDES1*-overexpressing plants.

Fig. S12. CRISPR/Cas9 characterization of *OsDES1*.

Fig. S13. Subcellular localization of the ΔOsDES1-GFP and NEMP-GFP fusion proteins.

Fig. S14. *In situ* analysis of *OsDES1* expression in longitudinal sections of the embryo sacs.

Fig. S15. Cytokinin determination and cytokinin-related gene expression.

Table S1. Oligonucleotide primers used in this study.

Table S2. Statistical analysis of pollen tube growth observed in the ovule at 2 h after pollination in WT and *des1*.

erac506_suppl_Supplementary_Figures_S1-S15_Tables_S1-S2

## Data Availability

All data supporting the findings of this study are available within the paper and within its supplementary data published online.

## References

[CIT0001] Argueso CT , Ferreira FJ, Epple P, To JPC, Hutchison CE, Schaller GE, Dangl JL, Kieber JJ. 2012. Two-component elements mediate interactions between cytokinin and salicylic acid in plant immunity. PLoS Genetics 8, e1002448. 22291601 10.1371/journal.pgen.1002448 PMC3266875

[CIT0002] Awasthi A , Paul P, Kumar S, Verma SK, Prasad R, Dhaliwal HS. 2012. Abnormal endosperm development causes female sterility in rice insertional mutant *OsAPC6*. Plant Science 183, 167–174. 22195590 10.1016/j.plantsci.2011.08.007

[CIT0003] Bao SG , Shen GS, Li GC, Liu ZK, Arif M, Wei QQ, Men SZ. 2019. The Arabidopsis nucleoporin NUP1 is essential for megasporogenesis and early stages of pollen development. Plant Cell Reports 38, 59–74. 30341574 10.1007/s00299-018-2349-7

[CIT0004] Bencivenga S , Simonini S, Benkova E, Colombo L. 2012. The transcription factors BEL1 and SPL are required for cytokinin and auxin signaling during ovule development in *Arabidopsis*. The Plant Cell 24, 2886–2897. 22786869 10.1105/tpc.112.100164 PMC3426121

[CIT0005] Berger F. 2011. Imaging fertilization in flowering plants, not so abominable after all. Journal of Experimental Botany 62, 1651–1658. 20952626 10.1093/jxb/erq305

[CIT0006] Boateng KA , Yang X, Dong F, Owen HA, Makaroff CA. 2008. *SWI1* is required for meiotic chromosome remodeling events. Molecular Plant 1, 620–633. 19825567 10.1093/mp/ssn030

[CIT0007] Chang YX , Gong L, Yuan WY, Li XW, Chen GX, Li XH, Zhang QF, Wu CY. 2009. Replication protein A (RPA1a) is required for meiotic and somatic DNA repair but is dispensable for DNA replication and homologous recombination in rice. Plant Physiology 151, 2162–2173. 19812186 10.1104/pp.109.142877 PMC2785997

[CIT0008] Chen YH , Li HJ, Shi DQ, Yuan L, Liu J, Sreenivasan R, Baskar R, Grossniklaus U, Yang WC. 2007. The central cell plays a critical role in pollen tube guidance in *Arabidopsis*. The Plant Cell 19, 3563–3577. 18055609 10.1105/tpc.107.053967 PMC2174880

[CIT0009] Cheng CY , Mathews DE, Schaller GE, Kieber JJ. 2013. Cytokinin-dependent specification of the functional megaspore in the Arabidopsis female gametophyte. The Plant Journal 73, 929–940. 23181607 10.1111/tpj.12084

[CIT0010] Chettoor AM , Evans MMS. 2015. Correlation between a loss of auxin signaling and a loss of proliferation in maize antipodal cells. Frontiers in Plant Science 6, 187. 25859254 10.3389/fpls.2015.00187 PMC4374392

[CIT0011] Christensen CA , Subramanian S, Drews GN. 1998. Identification of gametophytic mutations affecting female gametophyte development in *Arabidopsis*. Developmental Biology 202, 136–151. 9758709 10.1006/dbio.1998.8980

[CIT0012] Demesa-Arévalo E , Vielle-Calzada JP. 2013. The classical arabinogalactan protein AGP18 mediates megaspore selection in *Arabidopsis*. The Plant Cell 25, 1274–1287. 23572547 10.1105/tpc.112.106237 PMC3663267

[CIT0013] Dresselhaus T , Sprunck S, Wessel GM. 2016. Fertilization mechanisms in flowering plants. Current Biology 26, R125–R139. 26859271 10.1016/j.cub.2015.12.032 PMC4934421

[CIT0014] Drews GN , Koltunow AM. 2011. The female gametophyte. The Arabidopsis Book 9, e0155. 22303279 10.1199/tab.0155 PMC3268550

[CIT0015] Drews GN , Lee D, Christensen CA. 1998. Genetic analysis of female gametophyte development and function. The Plant Cell 10, 5–17. 9477569 10.1105/tpc.10.1.5 PMC143932

[CIT0016] Gigliotti S , Callaini G, Andone S, Riparbelli MG, Pernas-Alonso R, Hoffmann G, Graziani F, Malva C. 1998. *Nup154*, a new *Drosophila* gene essential for male and female gametogenesis is related to the *Nup155* vertebrate nucleoporin gene. Journal of Cell Biology 142, 1195–1207. 9732281 10.1083/jcb.142.5.1195 PMC2149350

[CIT0017] Heng YQ , Wu CY, Long Y, et al. 2018. OsALMT7 maintains panicle size and grain yield in rice by mediating malate transport. The Plant Cell 30, 889–906. 29610210 10.1105/tpc.17.00998 PMC5969278

[CIT0018] Heydlauff J , Groß-Hardt R. 2014. Love is a battlefield: programmed cell death during fertilization. Journal of Experimental Botany 65, 1323–1330. 24567492 10.1093/jxb/eru030

[CIT0019] Higashiyama T , Yang WC. 2017. Gametophytic pollen tube guidance: attractant peptides, gametic controls, and receptors. Plant Physiology 173, 112–121. 27920159 10.1104/pp.16.01571 PMC5210755

[CIT0020] Huang X , Peng X, Sun MX. 2017. *OsGCD1* is essential for rice fertility and required for embryo dorsal-ventral pattern formation and endosperm development. New Phytologist 215, 1039–1058. 28585692 10.1111/nph.14625

[CIT0021] Hwang I , Sheen J, Muller B. 2012. Cytokinin signaling setworks. Annual Review of Plant Biology 63, 353–380. 10.1146/annurev-arplant-042811-105503 22554243

[CIT0022] Jefferson RA. 1989. The GUS reporter gene system. Nature 342, 837–838. 2689886 10.1038/342837a0

[CIT0023] Jones-Rhoades MW , Borevitz JO, Preuss D. 2007. Genome-wide expression profiling of the *Arabidopsis* female gametophyte identifies families of small, secreted proteins. PLoS Genetics 3, 1848–1861. 17937500 10.1371/journal.pgen.0030171 PMC2014789

[CIT0024] Kou YJ , Chang YX, Li H, Xiao JH, Wang SP. 2012. The rice *RAD51C* gene is required for the meiosis of both female and male gametocytes and the DNA repair of somatic cells. Journal of Experimental Botany 63, 5323–5335. 22859673 10.1093/jxb/ers190 PMC3431001

[CIT0025] Kouchi H , Hata S. 1993. Isolation and characterization of novel nodulin cDNAs representing genes expressed at early stages of soybean nodule development. Molecular and General Genetics 238, 106–119. 7683079 10.1007/BF00279537

[CIT0026] Kumar M , Basha PO, Puri A, Rajpurohit D, Randhawa GS, Sharma TR, Dhaliwal HS. 2010. A candidate gene *OsAPC6* of anaphase-promoting complex of rice identified through T-DNA insertion. Functional & Integrative Genomics 10, 349–358. 20091079 10.1007/s10142-009-0155-6

[CIT0027] Kurakawa T , Ueda N, Maekawa M, Kobayashi K, Kojima M, Nagato Y, Sakakibara H, Kyozuka J. 2007. Direct control of shoot meristem activity by a cytokinin-activating enzyme. Nature 445, 652–655. 17287810 10.1038/nature05504

[CIT0028] Li DY , Huang ZY, Song SH, et al. 2016. Integrated analysis of phenome, genome, and transcriptome of hybrid rice uncovered multiple heterosis-related loci for yield increase. Proceedings of the National Academy of Sciences, USA 113, E6026–E6035. 10.1073/pnas.1610115113 PMC5068331 27663737

[CIT0029] Li SC , Li WB, Huang B, et al. 2013. Natural variation in *PTB1* regulates rice seed setting rate by controlling pollen tube growth. Nature Communication 4, 2793. 10.1038/ncomms3793 24240868

[CIT0030] Li N , Zhang DS, Liu HS, et al. 2006. The rice tapetum degeneration retardation gene is required for tapetum degradation and anther development. The Plant Cell 18, 2999–3014. 17138695 10.1105/tpc.106.044107 PMC1693939

[CIT0031] Li HJ , Zhu SS, Zhang MX, Wang T, Liang L, Xue Y, Shi DQ, Liu J, Yang WC. 2015. Arabidopsis CBP1 is a novel regulator of transcription initiation in central cell-mediated pollen tube guidance. The Plant Cell 27, 2880–2893. 26462908 10.1105/tpc.15.00370 PMC4682316

[CIT0032] Lieber D , Lora J, Schrempp S, Lenhard M, Laux T. 2011. *Arabidopsis WIH1* and *WIH2* genes act in the transition from somatic to reproductive cell fate. Current Biology 21, 1009–1017. 21658947 10.1016/j.cub.2011.05.015

[CIT0033] Liu CZ , Xue ZH, Tang D, Shen Y, Shi WQ, Ren LJ, Du GJ, Li YF, Cheng ZK. 2018. Ornithine δ-aminotransferase is critical for floret development and seed setting through mediating nitrogen reutilization in rice. The Plant Journal 96, 842–854. 30144334 10.1111/tpj.14072

[CIT0034] Long YM , Zhao LF, Niu BX, et al. 2008. Hybrid male sterility in rice controlled by interaction between divergent alleles of two adjacent genes. Proceedings of the National Academy of Sciences, USA 105, 18871–18876. 10.1073/pnas.0810108105 PMC2596266 19033192

[CIT0035] Lu JY , Wang CL, Wang HY, et al. 2020. *OsMFS1*/*OsHOP2* complex participates in rice male and female development. Frontiers in Plant Science 11, 518. 32499797 10.3389/fpls.2020.00518 PMC7243175

[CIT0036] Manrique S , Friel J, Gramazio P, Hasing T, Ezquer I, Bombarely A. 2019. Genetic insights into the modification of the pre-fertilization mechanisms during plant domestication. Journal of Experimental Botany 70, 3007–3019. 31152173 10.1093/jxb/erz231

[CIT0037] Mao BG , Zheng WJ, Huang Z, et al. 2021. Rice MutLγ, the MLH1–LH3 heterodimer, participates in the formation of type I crossovers and regulation of embryo sac fertility. Plant Biotechnology Journal 19, 1443–1455. 33544956 10.1111/pbi.13563 PMC8313138

[CIT0038] Meng JG , Liang L, Jia PF, Wang YC, Li HJ, Yang WC. 2020. Integration of ovular signals and exocytosis of a Ca^2+^ channel by MLOs in pollen tube guidance. Nature Plants 6, 143–153. 32055051 10.1038/s41477-020-0599-1

[CIT0039] Miao J , Guo D, Zhang J, Huang Q, Qin G, Zhang X, Wan J, Gu H, Qu LJ. 2013. Targeted mutagenesis in rice using CRISPR-Cas system. Cell Research 23, 1233–1236. 23999856 10.1038/cr.2013.123 PMC3790239

[CIT0040] Nakajima K. 2018. Be my baby: patterning toward plant germ cells. Current Opinion in Plant Biology 41, 110–115. 29223127 10.1016/j.pbi.2017.11.002

[CIT0041] Nonomura KI , Nakano M, Fukuda T, Eiguchi M, Miyao A, Hirochika H, Kurata N. 2004a. The novel gene *HOMOLOGOUS PAIRING ABERRATION IN RICE MEIOSIS1* of rice encodes a putative coiled-coil protein required for homologous chromosome pairing in meiosis. The Plant Cell 16, 1008–1020. 15031413 10.1105/tpc.020701 PMC412873

[CIT0042] Nonomura KI , Nakano M, Murata K, Miyoshi K, Eiguchi M, Miyao A, Hirochika H, Kurata N. 2004b. An insertional mutation in the rice *PAIR2* gene, the ortholog of *Arabidopsis ASY1*, results in a defect in homologous chromosome pairing during meiosis. Molecular Genetics and Genomics 271, 121–129. 14758540 10.1007/s00438-003-0934-z

[CIT0043] Pagnussat GC , Yu HJ, Sundaresan V. 2007. Cell-fate switch of synergid to egg cell in *Arabidopsis eostre* mutant embryo sacs arises from misexpression of the BEL1-like homeodomain gene *BLH1*. The Plant Cell 19, 3578–3592. 18055603 10.1105/tpc.107.054890 PMC2174879

[CIT0044] Pischke MS , Jones LG, Otsuga D, Fernandez DE, Drews GN, Sussman MR. 2002. An *Arabidopsis* histidine kinase is essential for megagametogenesis. Proceedings of the National Academy of Sciences, USA 99, 15800–15805. 10.1073/pnas.232580499 PMC137796 12426401

[CIT0045] Qin Y , Zhao LH, Skaggs MI, et al. 2014. ACTIN-RELATED PROTEIN6 regulates female meiosis by modulating meiotic gene expression in *Arabidopsis*. The Plant Cell 26, 1612–1628. 24737671 10.1105/tpc.113.120576 PMC4036575

[CIT0046] Rabiger DS , Drews GN. 2013. *MYB64* and *MYB119* are required for cellularization and differentiation during female gametogenesis in *Arabidopsis thaliana*. PLoS Genetics 9, e1003783. 24068955 10.1371/journal.pgen.1003783 PMC3778002

[CIT0047] Ray S , Park SS, Ray A. 1997. Pollen tube guidance by the female gametophyte. Development 124, 2489–2498. 9199374 10.1242/dev.124.12.2489

[CIT0048] Reiser L , Fischer RL. 1993. The ovule and the embryo sac. The Plant Cell 5, 1291–1301. 12271029 10.1105/tpc.5.10.1291 PMC160362

[CIT0049] Ren Y , Chen D, Li WJ, et al. 2019. OsSHOC1 and OsPTD1 are essential for crossover formation during rice meiosis. The Plant Journal 98, 315–328. 30589140 10.1111/tpj.14214

[CIT0050] Robinson-Beers K , Pruitt RE, Gasser CS. 1992. Ovule development in wild-type Arabidopsis and two female-sterile mutants. The Plant Cell 4, 1237–1249. 12297633 10.1105/tpc.4.10.1237 PMC160211

[CIT0051] Russell SD. 1996. Attraction and transport of male gametes for fertilization. Sexual Plant Reproduction 9, 337–342.

[CIT0052] Sankaranarayanan S , Higashiyama T. 2018. Capacitation in plant and animal fertilization. Trends in Plant Science 23, 129–139. 29170007 10.1016/j.tplants.2017.10.006

[CIT0053] Sheridan WF , Avalkina NA, Shamrov II, Batygina TB, Golubovskaya IN. 1996. The *mac1* gene: controlling the commitment to the meiotic pathway in maize. Genetics 142, 1009–1020. 8849906 10.1093/genetics/142.3.1009 PMC1207000

[CIT0054] Skinner DJ , Sundaresan V. 2018. Recent advances in understanding female gametophyte development [version 1; peer review: 2 approved]. F1000Research 7, 804. 10.12688/f1000research.14508.1 PMC6013762 29983913

[CIT0055] Song X , Qiu SQ, Xu CG, Li XH, Zhang QF. 2005. Genetic dissection of embryo sac fertility, pollen fertility, and their contributions to spikelet fertility of intersubspecific hybrids in rice. Theoretical and Applied Genetics 110, 205–211. 15672255 10.1007/s00122-004-1798-2

[CIT0056] Sun Y , Wang X, Pan L, Xie F, Dai B, Sun MX, Peng XB. 2021. Plant egg cell fate determination depends on its exact position in female gametophyte. Proceedings of the National Academy of Sciences, USA 118, e2017488118. 10.1073/pnas.2017488118 PMC7923378 33597298

[CIT0057] Tang Y , Dong QL, Wang TY, Gong L, Gu YN. 2022. PNET2 is a component of the plant nuclear lamina and is required for proper genome organization and activity. Developmental Cell 57, 19–31.e6. 34822788 10.1016/j.devcel.2021.11.002

[CIT0058] Tang Y , Huang A, Gu YN. 2020. Global profiling of plant nuclear membrane proteome in *Arabidopsis*. Nature Plants 6, 838–847. 32601417 10.1038/s41477-020-0700-9

[CIT0059] Tekleyohans DG , Nakel T, Groß-Hardt R. 2017. Patterning the female gametophyte of flowering plants. Plant Physiology 173, 122–129. 27920158 10.1104/pp.16.01472 PMC5210745

[CIT0060] Voinnet O , Rivas S, Mestre P, Baulcombe D. 2003. An enhanced transient expression system in plants based on suppression of gene silencing by the p19 protein of tomato bushy stunt virus. The Plant Journal 33, 949–956. 12609035 10.1046/j.1365-313x.2003.01676.x

[CIT0061] Wang N , Huang HJ, Ren ST, Li JJ, Sun Y, Sun DY, Zhang SQ. 2012. The rice wall-associated receptor-like kinase gene *OsDEES1* plays a role in female gametophyte development. Plant Physiology 160, 696–707. 22885936 10.1104/pp.112.203943 PMC3461549

[CIT0062] Wang TK , Li YX, Song SF, Qiu MD, Zhang LC, Li CX, Dong H, Li L, Wang J, Li L. 2021. EMBRYO SAC DEVELOPMENT 1 affects seed setting rate in rice by controlling embryo sac development. Plant Physiology 186, 1060–1073. 33734397 10.1093/plphys/kiab106 PMC8195536

[CIT0063] Wang CL , Wang Y, Cheng ZJ, et al. 2016. The role of OsMSH4 in male and female gamete development in rice meiosis. Journal of Experimental Botany 67, 1447–1459. 26712826 10.1093/jxb/erv540 PMC4762385

[CIT0064] Wang JY , Wang SZ, Hu K, et al. 2018. The kinase OsCPK4 regulates a buffering mechanism that fine-tunes innate immunity. Plant Physiology 176, 1835–1849. 29242377 10.1104/pp.17.01024 PMC5813524

[CIT0065] Xu Y , Wang FQ, Chen ZH, Wang J, Li WQ, Fan FJ, Tao YJ, Jiang YJ, Zhu QH, Yang J. 2020. CRISPR/Cas9-targeted mutagenesis of the *OsROS1* gene induces pollen and embryo sac defects in rice. Plant Biotechnology Journal 18, 1999–2001. 32323464 10.1111/pbi.13388 PMC7540548

[CIT0066] Xu Y , Yang J, Wang YH, et al. 2017. OsCNGC13 promotes seed-setting rate by facilitating pollen tube growth in stylar tissues. PLoS Genetics 13, e1006906. 28708858 10.1371/journal.pgen.1006906 PMC5533464

[CIT0067] Yadegari R , Drews GN. 2004. Female gametophyte development. The Plant Cell 16, S133–S141. 15075395 10.1105/tpc.018192 PMC2643389

[CIT0068] Yamaki S , Nagato Y, Kurata N, Nonomura KI. 2011. Ovule is a lateral organ finally differentiated from the terminating floral meristem in rice. Developmental Biology 351, 208–216. 21146515 10.1016/j.ydbio.2010.12.006

[CIT0069] Yang HJ , Iwamoto M, Hiraoka Y, Haraguchi T. 2017. Function of nuclear membrane proteins in shaping the nuclear envelope integrity during closed mitosis. Journal of Biochemistry 161, 471–477. 28398483 10.1093/jb/mvx020

[CIT0070] Yang WC , Ye D, Xu J, Sundaresan V. 1999. The *SPOROCYTELESS* gene of *Arabidopsis* is required for initiation of sporogenesis and encodes a novel nuclear protein. Genes & Development 13, 2108–2117. 10465788 10.1101/gad.13.16.2108 PMC316961

[CIT0071] Yang JY , Zhao XB, Cheng K, et al. 2012. A killer-protector system regulates both hybrid sterility and segregation distortion in rice. Science 337, 1336–1340. 22984070 10.1126/science.1223702

[CIT0072] Yoo SD , Cho YH, Sheen J. 2007. *Arabidopsis* mesophyll protoplasts: a versatile cell system for transient gene expression analysis. Nature Protocols 2, 1565–1572. 17585298 10.1038/nprot.2007.199

[CIT0073] Yu XW , Zhao ZG, Zheng XM, et al. 2018. A selfish genetic element confers non-Mendelian inheritance in rice. Science 360, 1130–1132. 29880691 10.1126/science.aar4279

[CIT0074] Yuan WY , Li XW, Chang YX, Wen RY, Chen GX, Zhang QF, Wu CY. 2009. Mutation of the rice gene *PAIR3* results in lack of bivalent formation in meiosis. The Plant Journal 59, 303–315. 19392701 10.1111/j.1365-313X.2009.03870.x

[CIT0075] Zafar SA , Patil SB, Uzair M, et al. 2019. *DEGENERATED PANICLE AND PARTIAL STERILITY 1* (*DPS1*) encodes a cystathionine β-synthase domain containing protein required for anther cuticle and panicle development in rice. New Phytologist 225, 356–375. 31433495 10.1111/nph.16133

[CIT0076] Zeng YX , Hu CY, Lu YG, Li JQ, Liu XD. 2009. Abnormalities occurring during female gametophyte development result in the diversity of abnormal embryo sacs and leads to abnormal fertilization in *indica*/*japonica* hybrids in rice. Journal of Integrative Plant Biology 51, 3–12. 19166488 10.1111/j.1744-7909.2008.00733.x

[CIT0077] Zhang DB , Luo X, Zhu L. 2011. Cytological analysis and genetic control of rice anther development. Journal of Genetics and Genomics 38, 379–390. 21930097 10.1016/j.jgg.2011.08.001

[CIT0078] Zhang K , Song Q, Wei Q, Wang CC, Zhang LW, Xu WY, Su Z. 2016. Down-regulation of *OsSPX1* caused semi-male sterility, resulting in reduction of grain yield in rice. Plant Biotechnology Journal 14, 1661–1672. 26806409 10.1111/pbi.12527 PMC5066639

[CIT0079] Zhang DB , Wilson ZA. 2009. Stamen specification and anther development in rice. Chinese Science Bulletin 54, 2342–2353.

[CIT0080] Zhao ZG , Jiang L, Zhang WW, Yu CY, Zhu SS, Xie K, Tian H, Liu LL, Ikehashi H, Wan JM. 2007. Fine mapping of *S31*, a gene responsible for hybrid embryo-sac abortion in rice (*Oryza sativa* L.). Planta 226, 1087–1096. 17549514 10.1007/s00425-007-0553-8

[CIT0081] Zhou SR , Wang Y, Li WC, et al. 2011. *Pollen semi-sterility1* encodes a kinesin-1-like protein important for male meiosis, anther dehiscence, and fertility in rice. The Plant Cell 23, 111–129. 21282525 10.1105/tpc.109.073692 PMC3051251

[CIT0082] Zuo JR , Li JY. 2014. Molecular genetic dissection of quantitative trait loci regulating rice grain size. Annual Review of Genetics 48, 99–118. 10.1146/annurev-genet-120213-092138 25149369

