# Supplementary material for: Mutation of DEFECTIVE EMBRYO SAC1 results in a low seed-setting rate in rice by regulating embryo sac development
Source: J Exp Bot. 2023 Jan 18;74(5):1501–16. doi: 10.1093/jxb/erac506 (PMC10010608; doi:10.1093/jxb/erac506)
Supplement: erac506_suppl_Supplementary_Figures_S1-S15_Tables_S1-S2 [file erac506_suppl_supplementary_figures_s1-s15_tables_s1-s2.pdf]

Supplementary figures

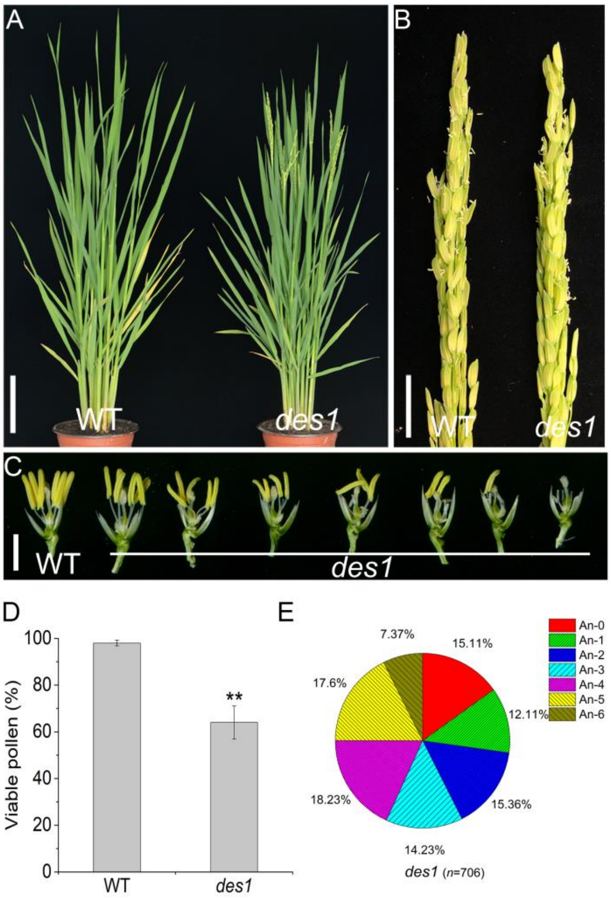

Supplementary Fig. S1. Phenotypic analysis in WT and *des1*. (A) WT and *des1* plants at the heading stage. Scale bar represents 20 cm. (B) Panicles of WT and *des1* plants at anthesis stage. Scale bar represents 2 cm. (C) Florets of WT and *des1*. The number of stamen is six in WT, and varied between zero to six in *des1*. The palea and lemma were removed for observation. Scale bar represents 2.5 mm. (D) Statistical analysis of viable pollen in WT and *des1*. Data are means ± SD (n = 3). (E) Frequency of stamen numbers in mature spikelets of *des1*. n, spikelet number. An 0-6 represents stamen numbers, respectively.

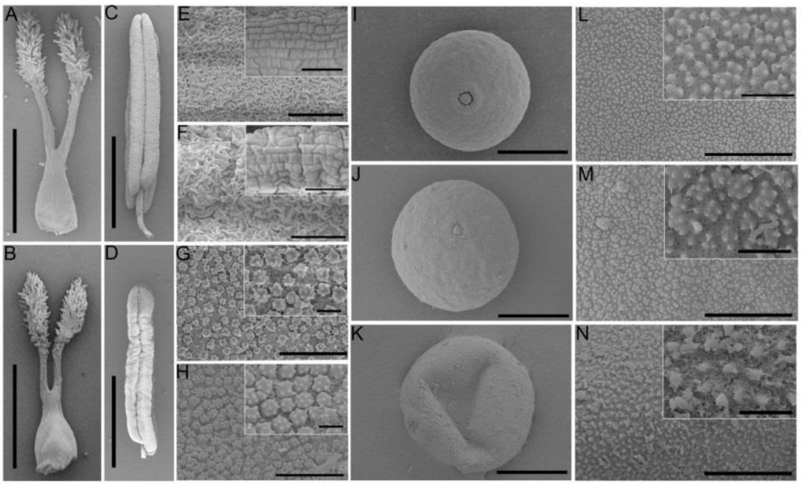

Supplementary Fig. S2. Scanning electron microscopy (SEM) observation of the mature pistils, anthers and pollen grains in WT and *des1*. Comparisons of SEM observations in WT (A, C, E, G, I, L) and *des1* (B, D, F, H, J, K, M, N) mature pistils (A, B), anthers (C, D), anther epidermis (E, F), anther inner surfaces (G, H), pollen grains, (I, J, K), and pollen exine (L, M, N). Scale bars represent 1 mm in (A-D), 10 μm in (E, F), 100 μm in the enlarged of (E, F), 5 μm in (G, H, L, M, N), 1 μm in the enlarged of (G, H, L, M, N), and 20 μm in (I, J, K).

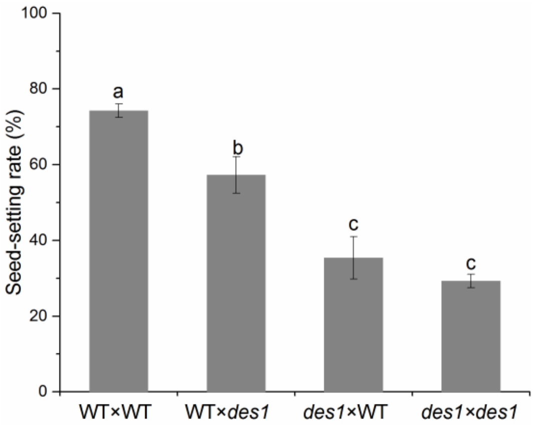

Supplementary Fig. S3. Statistical data of the seed-setting rate of the reciprocal crosses. Data are Means ± SD from 3 replicates with > 40 emasculated spikelets per replicate, and different letters indicate significant differences as determined by Duncan's test ($P < 0.05$).

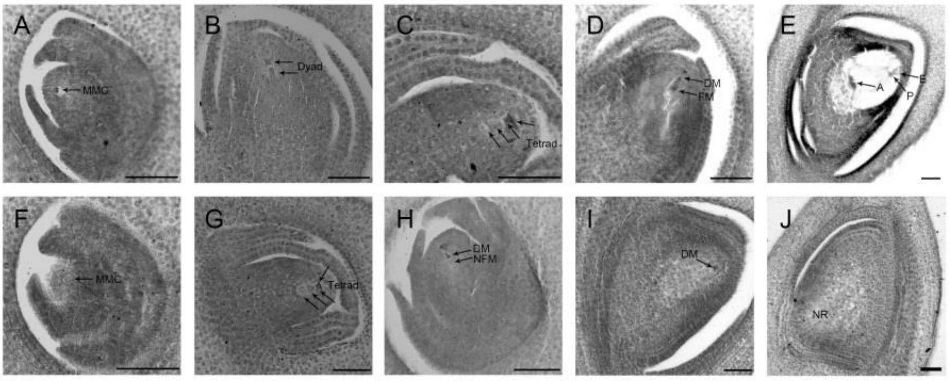

Supplementary Fig. S4. Paraffin section analysis of the embryo sac development in WT and *des1*. (A-E) Images of embryo sacs in WT at megasporocyte stage (A), dyad (B), tetrad (C), functional megaspore formation stage (D) and mature embryo sac stage (E), respectively. (F-H) Images of embryo sacs in *des1* at megasporocyte formation stage (F), tetrad (G) and functional megaspore formation stage (H), respectively. Part (I) shows that the so-called functional megaspore degenerated along with the other three megaspores in *des1*. Part (J) shows no embryo sac formation in *des1*. MMC, megaspore mother cell; DM, degenerated megaspore; FM, functional megaspore; A, antipodal cell; P, polar nucleus; E, egg cell; NFM, non-functional megaspore; NR, nucellar remnants. Bars = 50 μm.

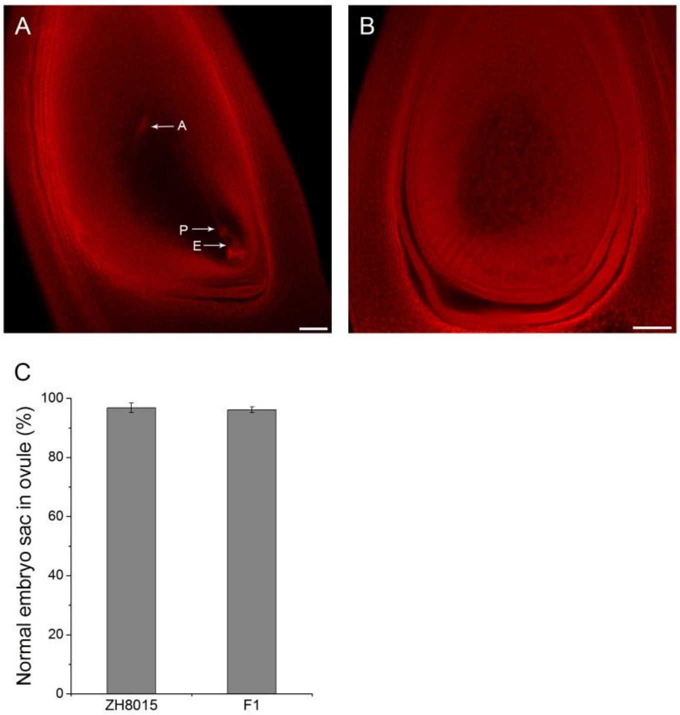

Supplementary Fig. S5. Microscopic observations of mature embryo sacs observations in ZH8015 and F_1 plants. (A) The normal embryo sac at maturity. (B) The degenerated embryo sac at maturity. (C) Statistical analysis of normal mature embryo sac formation in ZH8015 and F_1 plants. A, antipodal cell; P, polar nucleus; E, egg cell; S, synergid cell. Scale bars represent 50 μm. (Ovule number: ZH8015: 169; F_1: 179).

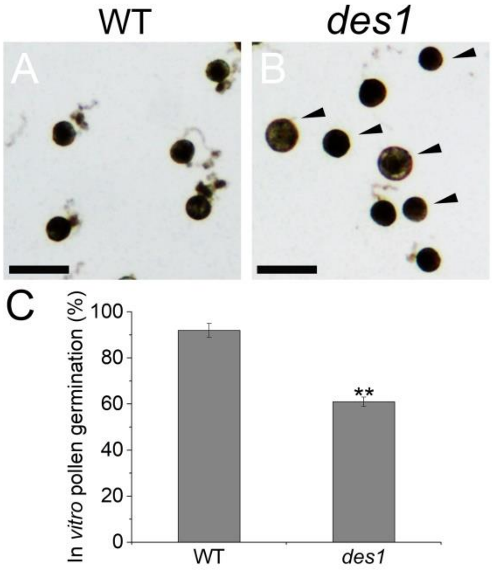

Supplementary Fig. S6. In vitro pollen germination assay. (A, B) Germination of WT and *des1* pollen grains *in vitro*. Scale bars represent 10 μm. (C) Percentage of WT and *des1* pollen grain germination *in vitro*. Data are means ± SD (n = 3). Arrows indicate the pollen grains that do not germinate *in vitro*.

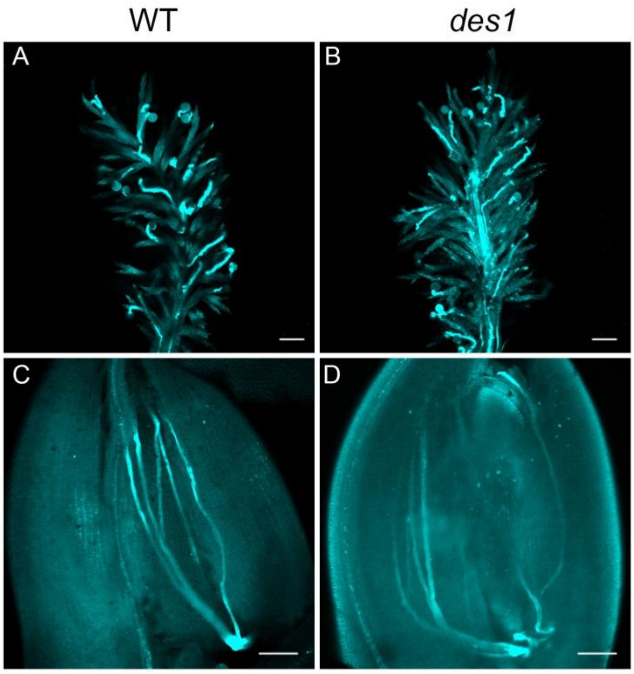

Supplementary Fig. S7. Pollen germination on the stigma and pollen tube growth in WT and *des1*. (A, B) Aniline blue staining of pollen germination on the stigma at 2 h after pollination in WT (A) and *des1* (B). (C, D) Aniline blue staining of pollen tube growth in the ovule at 2 h after pollination in WT (C) and *des1* (D). Scale bars represent 100 μm.

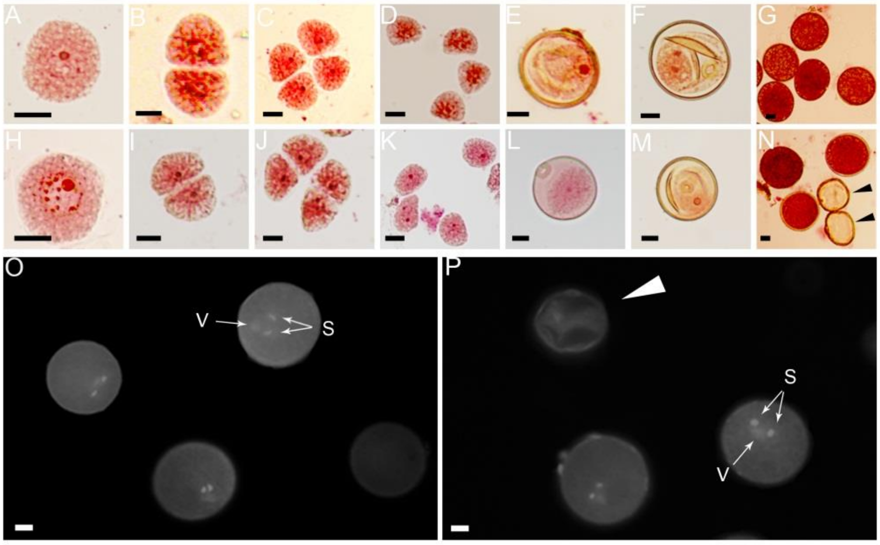

Supplementary Fig. S8. Male gametogenesis in WT and *des1* shown by aceto-carmine and DAPI staining. (A-N) The process of microspore development in WT (A-G) and *des1* (H-N) shown by aceto-carmine staining. (O, P) The process of microspore development in WT (O) and *des1* (P) shown by DAPI staining. Arrows indicate the aborted microspores. (A, H) The pollen mother cell differentiation stage; (B, I) the dyad stage; (C, J) the tetrad stage; (D, K) the early microspore stage; (E, L) the uninucleate stage; (F, M) the bicellular stage; (G, N) the mature pollen stage; (O, P) the tricellular stage. S, sperm nuclei; V, vegetative nuclei. Scale bars represent 10 μm in (A-P).

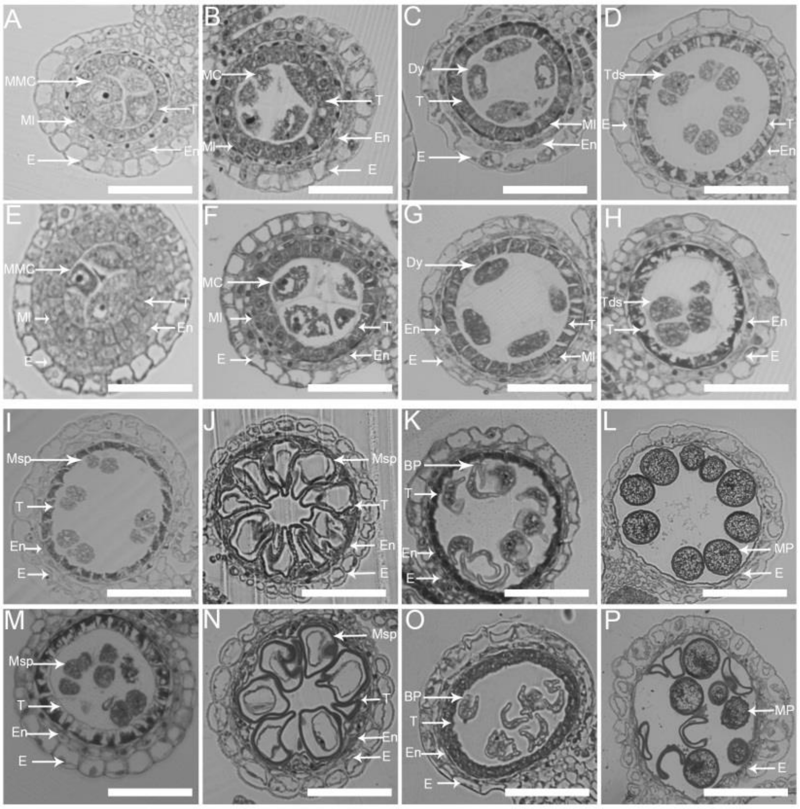

Supplementary Fig. S9. Transverse section observations of WT and *des1* anthers at various developmental stages. Transverse section of WT (A-D), (I-L) and *des1* (E-H), (M-P) anthers. The transverse sections (A-P) were stained with 0.25% toluidine blue. (A, E) Microspore mother cell stage; (B, F) the PMC pre-meiosis stage; (C, G) the dyad stage; (D, H) the tetrad stage; (I, M) the early microspore stage; (J, N) the vacuolated microspore stage; (K, O) the bicellular pollen stage; (L, P) the mature pollen stage. MMC, microspore mother cell; PMC, pollen mother cell; T, tapetum; ML, middle layer; En, endothecium; E, epidermis; MC, meiotic cell; Dy, dyad cell; Tds, tetrads; Msp, microspore parietal cell; Bp, bicellular pollen; Mp, mature pollen. Scale bars represent 25 μm in A-P.

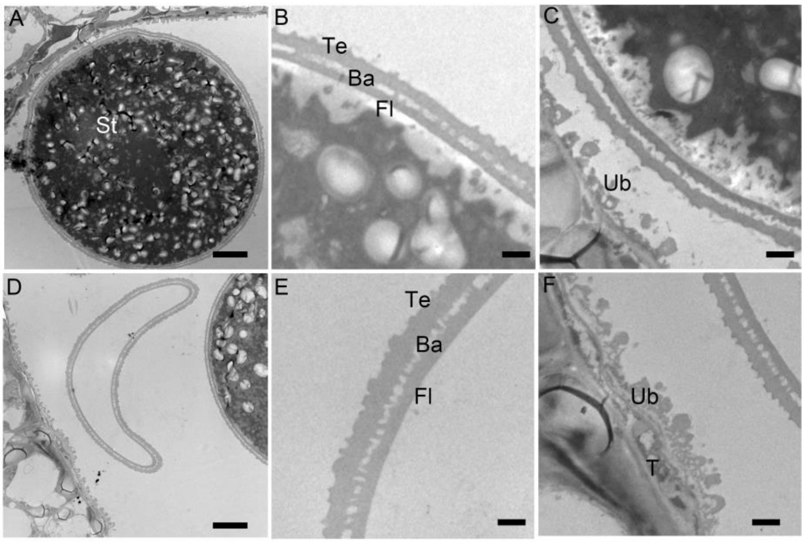

Supplementary Fig. S10. Transmission electron microscopy (TEM) observations of mature anthers in WT and *des1*. Comparisons of TEM observations in the mature WT (A-C) and *des1* (D-F) pollen (A, D), pollen wall (B, E),

and tapetum (C, F). St, Starch granules; Fl, Foot layer; Ba, Baculum; Te, Tectum; Ub, Ubisch body; T, tapetum. Scale bars represent 5 μm in (A, D), and 1 μm in (B, C, E, F).

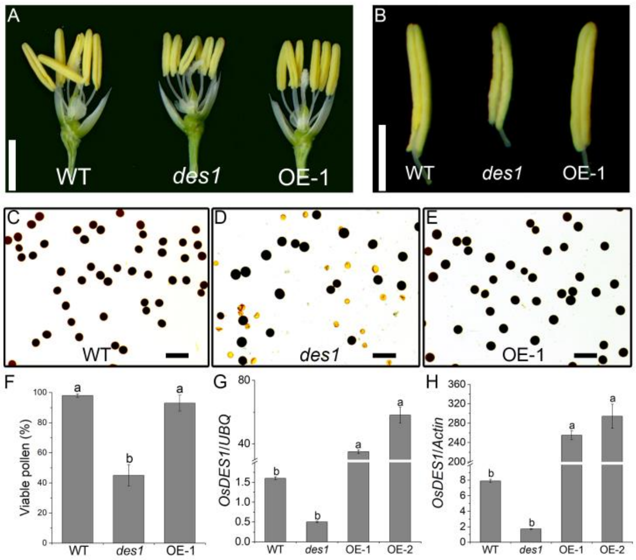

Supplementary Fig. S11. The anthers and pollen grains of WT, *des1*, and *OsDES1*-over-expression plants. (A, B) Comparison of the spikelets (A) and anthers (B) in WT, *des1*, and OE-1 plants. The palea and lemma were removed for observation. (C-E) I_2-KI staining of pollen grains in WT, *des1*, and OE-1. (F) Statistical analysis of pollen viability in WT, *des1*, and OE-1 plants. (G, H) Relative gene expression analysis in WT, *des1*, and over-expression plants. Data are means ± SD (n = 3). Different letters indicate significant differences as determined by Duncan's test ($P < 0.05$).Scale bars represent 0.25 cm in (A), 0.125 cm in (B), and 25 μm in (C-E).

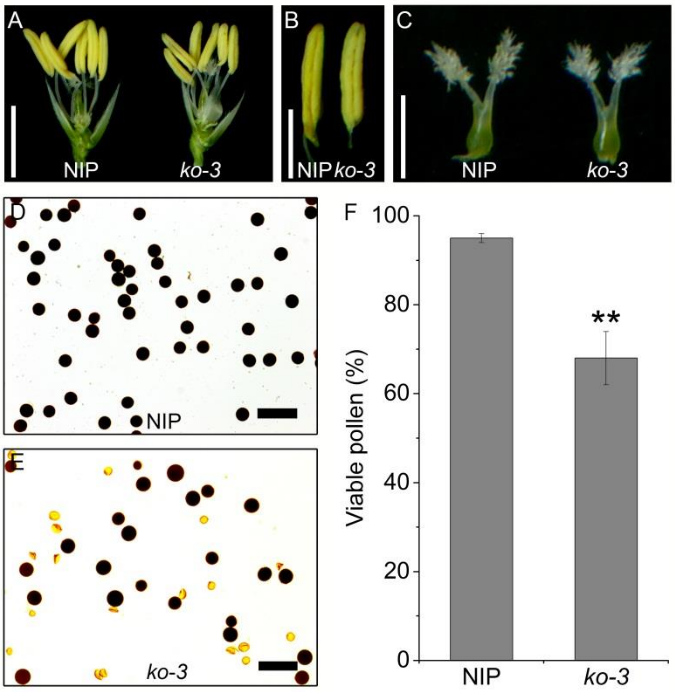

Supplementary Fig. S12. CRISPR-Cas9 characterization of *OsDES1*. (A) Comparison of NIP and *ko-3* spikelets. The palea and lemma were removed for observation. Scale bar represents 2.5 mm. (B) Anthers of NIP and *ko-3*.

Scale bar represents 1.25 mm. (C) Pistils of NIP and *ko-3*. Scale bar represents 1.25 mm. (D, E) I_2-KI staining of pollen grains from NIP and *ko-3* plants. Scale bars represent 25 μm. (F) Statistical analysis of the percentage of viable pollen in NIP and *ko-3* plants. Data are means ± SD (n = 3).

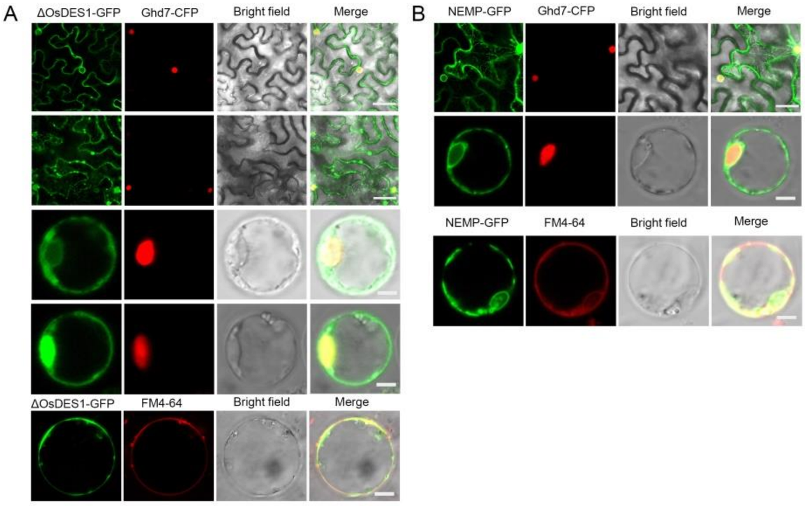

Supplementary Fig. S13. Subcellular localization of the △OsDES1-GFP and NEMP-GFP fusion proteins. A. Subcellular localization of the △OsDES1-GFP in *N. benthamiana* leaf epidermal cells and rice protoplasts. △OsDES1 represents the mutant OsDES1 protein. B. Subcellular localization of the NEMP-GFP in *N. benthamiana* leaf epidermal cells and rice protoplasts. Nuclear envelop membrane protein (NEMP) domain was located at amino acid residues 157-403. The Ghd7-CFP fusion protein was used as the nuclear marker. The plasma membrane was stained with FM4-64. Scale bars represent 50 μm and 5 μm.

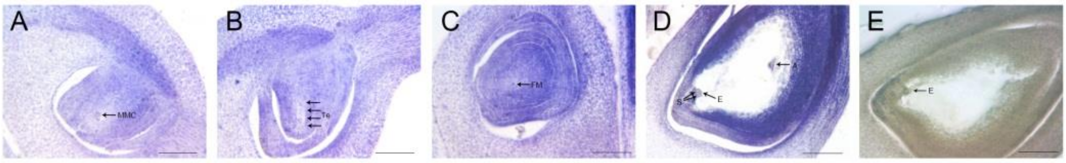

Supplementary Fig. S14. *In situ* analysis of *OsDES1* expression in longitudinal sections of the embryo sacs. (A) Megasporocyte stage. (B) Tetrad. (C) Functional megaspore formation stage. (D) Mature embryo sac stage. (E) Negative controls with the sense probe of the embryo sac at maturity. MMC, megaspore mother cell; Te, Tetrad; FM, functional megaspore; A, antipodal cell; S, synergid cell; E, egg cell. Scale bar represents 100 μm.

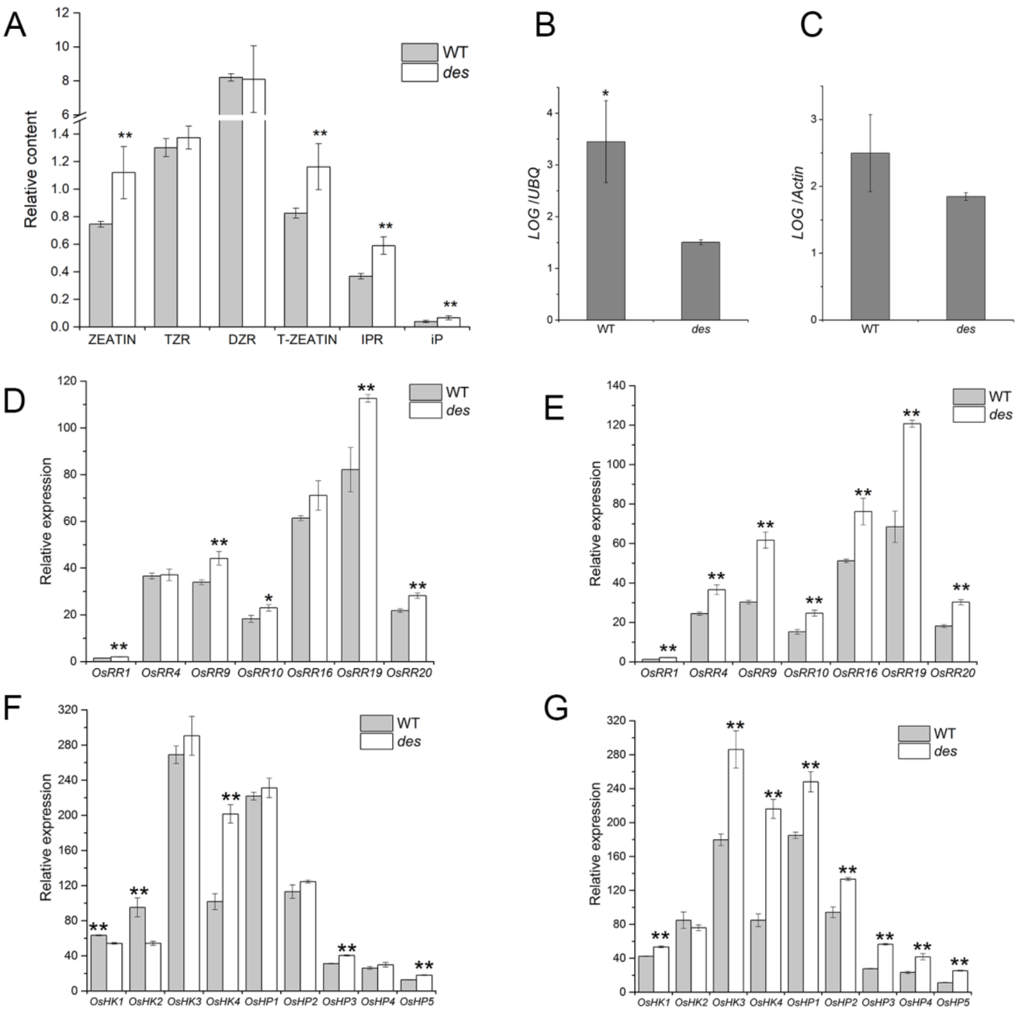

Supplementary Fig. S15. Cytokinin determination and cytokinin-related genes expression. (A) Statistical analysis of cytokinin contents. (B) and (C) *LOG* expression levels of the pistils of WT and *des1* at maturity. The *UBQ* and *Actin* gene were used as the inter controls, respectively. (D-G) Relative expression levels of cytokinin signal transduction-related genes between the pistils of WT and *des1* at maturity. The *UBQ* and *Actin* gene were used as the inter controls, respectively. Data are the means ± SD of three independent biological replicates. Asterisks indicate a significant difference by Student's *t*-test (* $P < 0.05$, ** $P < 0.01$).

Supplementary tables

Supplementary Table S1. Statistical analysis of pollen tube growth observed in the ovule at 2 h after pollination in WT and *des1*.

**Line**	**WT**	***des1***
Total	44	51
Ovules with pollen tube	37	41
Percent	84%	80%

Observation of pollen tube growth was defined as when at least one pollen tube in the ovule reached the micropyle at 2 h.

Supplementary Table S2. Oligonucleotide primers used in this study.

**Primer**	**Primer sequences（5'-3'）**	**Purpose**
3-24-F	GCAACCCTTTCTTCCTCCTC	Fine mapping
3-24-R	CCAAGGAGAGCGCACTAGC	

X55-F	AAGATTGAAGAAGCGGTCAAGC	
X55-R	GCTTGCATGCATAGATTTCTCC	
H2-F	CCTTGCTTCCCACCTTGA	
H2-R	TTGGTATTGCCGTTGCTT	
H35-F	CGAATAGGAACCGAGACT	
H35-R	TAAGGACGTGGGAGAGAG	
H58-F	ACCACCATACAGCACAGC	
H58-R	CATCACAAGTAGCAAGCC	
H30-F	CTGCCCTGGATACGTTAT	
H30-R	CCCTCGTGCTACTTTGAC	
3-31-F	ACTAGAGCACCCTCGCTGAG	
3-31-R	CTCAGCCACCCCATCAAC	
OsDES1-SeqF	AGGTGTATTCCTTCTCGTAAGTGTGA	Sequencing for mutation site
OsDES1-SeqR	GGATCATAACTGCAGAAATAATCAAG	
OsDES1GBD-1F	TTCTGCACTAGGTACCTGCAGATGCCACCACTCCACCGCCG	Overexpression
OsDES1GBD-1R	TCTTAGAATTCCCGGGGATCCTTAAAACAGTCCGAACAAACGTTTC	
1132-OsDES1-F	TCCCCCGGGCTGCAGGAATTCATGCCACCACTCCACCGC	
1132-OsDES1-R	GGTACCGGGCCCCCCCTCGAGAAACAGTCCGAACAAACGTTTCC	
1132-△OsDES1-F	TCCCCCGGGCTGCAGGAATTCATGCCACCACTCCACCGC	Subcellular localization
1132-△OsDES1-R	GGTACCGGGCCCCCCCTCGAGGGCTCCAGCTAAGATCACACTTAC	
1132NEMPGFP-F	TCCCCCGGGCTGCAGGAATTCGGAGGAAGAGTTCTTGCTTCACA	
1132NEMPGFP-R	GGTACCGGGCCCCCCCTCGAGTTGAGACAATGTCTTCCTTGACCG	
LOG-AD-F	GTTCCAGATTACGCTGGATCCATGGCAATGGAGGCTGCG	
LOG-AD-R	TTGATACCACTGCTTGGATCCTCAGGATGAGGTGATCCTGGTC	Yeast two hybrid
OsDES1-BD-F	CAAAATATCTGCAATGGCCATTACGGCCATGCCACCACTCCACCGC	
OsDES1-BD-R	CGAATTCCTGCAGATGGCCGAGGCGGCCCCAAACAGTCCGAACAAACGTTTC	
GFP-OsDES1-F	GATGAACTATACAAAGGCGCGCCAATGCCACCACTCCACCGC	
GFP-OsDES1-R	CGATCGGGGAAATTCGAGCTCTTAAAACAGTCCGAACAAACGTTTCC	Co-IP
Myc-LOG-F	AGAGGACTTGAATTCGGTACCCATGGCAATGGAGGCTGCG	
Myc-LOG-R	GTCCTAGGCTACGTAGGATCCTCAGGATGAGGTGATCCTGGTC	
nLUC-OsDES1-F	GAGCTCGGTACCCGGGGATCCATGCCACCACTCCACCGC	
nLUC-OsDES1-R	GCGTACGAGATCTGGTCGACAAACAGTCCGAACAAACGTTTCC	Luciferase complementation imaging assay
cLUC-LOG-F	GGGGCGGTACCCGGGGATCCATGGCAATGGAGGCTGCG	
cLUC-LOG-R	CGAAAGCTCTGCAGGTCGACTCAGGATGAGGTGATCCTGGTC	
GUS-1F	CAGTGAATTCGATAATAATTCGGCCCAGGC	GUS
GUS-1R	CGATCCATGGCGCGGCGGCGATTGAGGGAT	
OsDES1-ZF	AGCTATGAAACAACTGGTCTCA	
OsDES1-ZR	CAGCTTAAAACAGTCCGAACAA	
LOG-F:	TCCTAGGCAGCTATAGTAGTAGG	
LOG-R:	TGTAAGATTGTTGTTCCGTTCG	qRT-PCR
OsHK1-F	GATGTACTTGATCGGGCTAAGA	
OsHK1-R	ATCACATCATCCATGAGAGACC	
OsHK2-F	CATTTGAGGATTTCACGGCTAG	
OsHK2-R	CTTTTGCTCAAACAACTCCCTT	

OsHK3-F	GTTTCATGGACATACAGATGCC	
OsHK3-R	ATAGCCATCCATTTCGCTTTTC	
OsHK4-F	CTGCTGTACACCAAGAGAGTAA	
OsHK4-R	CTCTCATTCAGATCGCATCCTC	
OsHP1-F	GACAGGATCATCAACGAGATCG	
OsHP1-R	CCTTTGAGCTGATGAACGTAGG	
OsHP2-F	GTGAAGAACACTTGCATTCAGT	
OsHP2-R	CACCAAATCCAAAGTCTTGAGG	
OsHP3-F	AGGTGGGATACATATATGCAGC	
OsHP3-R	GACATGCACTCATTCTTCATCC	
OsHP4-F	CAATTGAAAGGCAGCTGTTCTA	
OsHP4-R	CCTCTTCACTTTCTGGAAGGAT	
OsHP5-F	GAAGAATGAGTGCTCTGTGTTC	
OsHP5-R	CTTGAGCTCACTGCATAATCAC	
OsRR1-F	GTCATGTCGTCGGAAAACG	
OsRR1-R	CCTTGCTTTGAAGAGGCTTTAC	
OsRR4-F	CTCAAGAACTCGTCCTACCAAG	
OsRR4-R	CTCTTGAGCAGATCATACCCTG	
OsRR9-F	AACATATCTGTGCTAGCTTTGC	
OsRR9-R	AAAGAAGAGGTCTTGAGTAGCC	
OsRR10-F	GTAGCCTCTTCCTTCTGTTCTT	
OsRR10-R	AAAGAAGAGGTCTTGAGTAGCC	
OsRR16-F	CGTTGACATGTACAGTGGATTC	
OsRR16-R	GATAACTGGGTAAGTCCTCAGG	
OsRR19-F	GTTCTTGCAACTTCTCAACCAT	
OsRR19-R	TCTGTCTTAAGAACACCAGGTG	
OsRR20-F	GGAGAGACAAAGACTGTGATGA	
OsRR20-R	TACCGAACTTCCTCCTAACAAC	
UBQ-F	GCTCCGTGGCGGTATCAT	
UBQ-R	CGGCAGTTGACAGCCCTAG	
Actin-F	TGCTATGTACGTCGCCATCCAG	
Actin-R	AATGAGTAACCACGCTCCGTCA	
InOsDES1-F	GGGTCCTCGATTTCCGGATG	RNA *in situ* hybridization
InOsDES1-R	AGACTCGCTAAGAGTGTGCG	
